# Effects of Medium-Chain Versus Medium- and Long-Chain Triglycerides, Combined with Carotenoids, in a High-Fat Diet on Obese Mice

**DOI:** 10.3390/foods15132285

**Published:** 2026-06-25

**Authors:** Ruihong Ge, Keyu Tu, Jinyang Li, Liang Wu, Yongjian Ge, Yongkang Niu, Shiyu Chen, Qinglong Wu, Ruozhen Wang, Shiqing Chen, Yoong Junhao, Hui Wang

**Affiliations:** 1 School of Public Health, Shanghai Jiao Tong University, Shanghai 200025, China; 2 College of Basic Medical Sciences, Shanghai Jiao Tong University, Shanghai 200025, China; 3 Palm Oil Research and Technical Service Institute, Malaysian Palm Oil Board, Shanghai 201108, China

**Keywords:** medium- and long-chain triglycerides, carotenoids, obesity, lipid metabolism, gut microbiota

## Abstract

While medium-chain triglycerides (MCTs), medium- and long-chain triglycerides (MLCTs), and carotenoids individually possess anti-obesity properties, the synergistic metabolic regulatory effects of their combined intervention remain under-investigated. This study explored the effects of MCTs or structured MLCTs combined with natural carotenoids on high-fat diet (HFD)-induced obese mice. After establishing obesity in C57BL/6J mice using a 60% HFD, a ten-week intervention was conducted using 45% HFD containing 150 mg/kg carotenoids across three groups: MCT-C, MLCT-C, and a physical mixture of MCTs and long-chain triglycerides plus carotenoids (MCT+LCT-C), alongside a low-fat diet (LFD) control. Results showed that among the three HFD-fed intervention groups, the MCT-C group had the lowest body weight with significantly lower fat mass, fat pad coefficient, and adipocyte area, but higher liver coefficient and serum alanine aminotransferase levels compared to the LFD control group (*p* < 0.05). The MLCT-C and MCT+LCT-C groups exhibited higher body weight, white adipose tissue expansion, and adipocyte hypertrophy, with MCT+LCT-C showing the largest adipocyte volume and uniquely presenting hepatocyte necrosis, unlike other groups. Serum total cholesterol was lowest in MCT-C, while high-density lipoprotein cholesterol increased in MLCT-C and MCT+LCT-C. Notably, compared with the MCT+LCT-C group, MLCT-C demonstrated distinctly superior capabilities in maintaining gut microbiota homeostasis, as evidenced by enhanced community alpha diversity and significantly reduced the abundance of harmful *Pseudomonadota*, while preserving a highly comparable core functional profile. Collectively, these findings confirm that lipid structure differentially shapes gut microbiota and influences energy metabolism, providing a scientific basis for precision nutrition interventions.

## 1. Introduction

According to the World Health Organization, obesity is a pathological condition where excess fat accumulation impairs health [1]. Over the past few decades, rapid economic and social transformations have drastically altered lifestyles, leading to increased consumption of high-calorie, high-fat foods and a decline in physical activity. These influences have fueled the rapid escalation of obesity on a global scale. According to the WHO, over 890 million adults worldwide are currently classified as obese [1], and this number is expected to rise to 1.02 billion by 2030 [2]. Obesity and overweight constitute serious worldwide public health concerns [3], putting a large burden on healthcare systems. These conditions constitute major modifiable global mortality risk factors, demonstrating dose-dependent associations with chronic diseases, including but not limited to type-2 diabetes mellitus, cardiovascular pathologies, obstructive sleep apnea, and neoplastic conditions [4,5]. To address this pressing issue, the current global non-communicable disease control framework prioritizes reducing obesity prevalence to 2010 baseline levels by 2025 [6].

Approaches to tackle obesity include lifestyle modifications, medication, and operative procedures. While clinical treatments such as pharmacotherapy and surgery are effective for weight loss and maintaining long-term results, they are also associated with adverse effects, including nausea, diarrhea, drowsiness, dry mouth, headache, dizziness, gastrointestinal discomfort (e.g., gastric ulcers) [7]. Although lifestyle changes have demonstrated benefits in lowering complication rates and mortality, maintaining a balanced and sensible diet remains fundamental to effective weight control and to decreasing obesity-related disease burden. Within this context, nutrition and natural ingredients play a pivotal role in both preventing and treating obesity.

MCTs are a specific type of dietary fat formed by a glycerol backbone esterified with three medium-chain fatty acids (MCFAs), namely caprylic acid (C8:0), capric acid (C10:0), and lauric acid (C12:0) [8]. Commercial MCT oils are typically produced by fractionating and re-esterifying coconut oil or palm kernel oil to enrich C8:0 and C10:0. MCTs have been reported to confer various health benefits and play roles in metabolic regulation and brain function [8,9].

MCTs exhibit anti-obesity effects, such as reducing body weight gain and lipid accumulation [10]. Continuous consumption of a high-MCT diet attenuates obesity and ameliorates insulin resistance in mice that are obese due to diet [11]. The known mechanisms by which MCTs alleviate obesity include changes in gut microbiota, increased gene expression of peroxisome proliferator-activated receptor gamma (PPAR-γ) and adiponectin in adipose tissue, enhanced hepatic β-oxidation, activation of brown adipocytes, and increased energy expenditure [12].

MCFAs derived from MCTs are rapidly metabolized as fuel in the liver because MCFAs with chain lengths shorter than those of long-chain fatty acids (LCFAs) can pass through the portal vein after intestinal absorption due to their higher water solubility. Thus, MCFAs are not accumulated as fat but are efficiently converted into energy for immediate organ utilization. Additionally, MCT intake increases energy sources through the rapid formation of ketone bodies, as excessive acetyl-CoA is generated by metabolizing MCFAs in the liver [13].

In recent years, research on MCTs and MLCTs has extended beyond their role as rapid oxidative energy sources. Recent studies demonstrate that MCTs and their intestinal metabolic derivatives can act as crucial signaling molecules, deeply participating in the remodeling of host lipid metabolic networks and energy homeostasis. Specifically, they may be able to modulate PPAR-α signaling to sustain lipid oxidation in obesity [14], upregulate the AMPK signaling pathway to activate brown adipose tissue [15], and regulate the PI3K/AKT and PPAR-γ pathways to ameliorate glucose and lipid metabolism disorders [16]. These findings provide a novel theoretical basis for their combined application with carotenoids.

MCTs are used in the food and pharmaceutical industries, applied for reducing body fat accumulation, promoting weight loss, improving nutritional status, and regulating nervous system diseases. However, due to the lack of LCFAs, MCTs have significant limitations in their physicochemical and metabolic properties, such as low smoke point, easy foaming, and rapid ketone body production, which restrict their use in food formulations [17]. Additionally, from a nutritional perspective, MCTs cannot provide sufficient LCFAs, which are crucial for fatty acid transport to maintain normal physiological functions of the body [18].

MLCTs represent a novel class of functional structured lipids, synthesized through the esterification of glycerol with three fatty acid molecules, comprising both LCFAs and MCFAs [19]. Naturally occurring MLCTs are uncommon, with primary sources being mammalian milk fats (e.g., from humans, cattle, and sheep) and a few plant-based oils such as palm kernel and coconut oil. Current synthetic strategies for MLCT production encompass direct esterification, transesterification, acidolysis, and a two-step enzymatic methodology [17,20]. MLCTs synergize the nutritional and metabolic advantages of MCTs and long-chain triglycerides (LCTs) while addressing their individual limitations [21]. Specifically, MLCTs compensate for the deficiency of essential fatty acids in MCTs and counteract the fat deposition linked to LCFAs, while simultaneously offering the benefit of rapid energy delivery. The specific distribution of fatty acids on the glycerol backbone of MLCTs determines their metabolic advantages over simple physical mixtures. Pancreatic lipase regioselectively hydrolyzes the sn-1 and sn-3 positions, releasing MCFAs for direct transport via the portal vein and subsequent hepatic oxidation. Simultaneously, the LCFAs retained at the sn-2 position form 2-monoacylglycerols (2-MAG), which are crucial for stabilizing mixed micelles and facilitating chylomicron assembly within enterocytes. Notably, approximately 75% of the sn-2 fatty acids are retained in the newly synthesized chylomicrons, dictating their structural features and influencing their subsequent metabolic fate. In contrast, physical mixtures are theoretically highly susceptible to asynchronous digestion and phase separation within the gastrointestinal tract, thereby compromising the delivery efficiency of lipophilic substances [17,20]. Furthermore, MLCTs facilitate the synchronized transport of fatty acids, thereby reducing the risk of excessive ketone production [18]. In comparison to LCTs, MLCTs demonstrate greater effectiveness in nutritional metabolism, which includes inhibiting adipose tissue accumulation, reducing serum triglycerides and cholesterol levels, reducing protein breakdown, supporting nitrogen equilibrium, and enhancing immune function [22].

Accumulating evidence indicates that MLCTs and their hydrolytic metabolites, MCFAs, potentiate energy expenditure and attenuate adipogenesis through pleiotropic mechanisms. These encompass hepatic metabolic reprogramming [23], mitigation of insulin resistance [24], gut microbiota remodeling [25], and activation of β-oxidation pathways [20]. Preclinical investigations employing a C57BL/6J mouse model demonstrated that six-week dietary supplementation with MLCTs (95% purity) derived from *Camellia oleifera* oil and *Cinnamomum camphora* seed oil significantly reduced body mass index and adipose tissue accretion. The anti-obesity effect was facilitated by increased fecal lipid excretion and the upregulation of lipolytic enzymes, such as cAMP-dependent protein kinase A (PKA), hormone-sensitive lipase (HSL), and adipose triglyceride lipase (ATGL) [26].

Similarly, Sprague Dawley (SD) rats fed MLCTs showed lighter liver mass, lower plasma triglyceride and cholesterol levels, and reduced liver cholesterol levels compared to those fed regular edible oil. These findings suggest that a diet containing MLCTs effectively prevents obesity, reduces body fat and blood lipids, and improves liver lipid metabolism [27]. Additionally, in MLCTs, MCFAs contribute to reduced fat accumulation through multiple metabolic pathways, including increased energy expenditure, enhanced hepatic fatty acid β-oxidation, and appetite regulation. In addition, MCFAs are involved in the regulation of lipid synthesis–related gene expression, such as PPAR-α, whose activation is associated with increased fatty acid oxidation [28].

As fat-soluble pigments found in nature, carotenoids have attracted increasing attention for their anti-obesity effects. A growing body of preclinical evidence suggests that these compounds participate in the regulation of body fat accumulation through multiple molecular pathways, supporting their potential function in preventing and managing obesity. Endogenous metabolism of carotenoids and retinoids also plays a role in physiological processes governing adiposity. Animal studies indicate that both provitamin A and non-provitamin A carotenoids are capable of reducing fat mass [29,30,31,32]. In particular, provitamin A activity appears to underlie the anti-obesity effects of β-carotene reported in rodent studies, which is consistent with the established anti-adiposity properties of retinoids [33]. The amount and type of carotenoid precursors consumed can modulate the production of endogenous RAR/RXR ligands via BCO1 activity, with subsequent effects on lipid metabolic processes. These processes have a pronounced impact on obesity and diabetes [29,34].

Research confirms that MCTs, MLCTs, and carotenoids can independently inhibit fat accumulation, but their combined anti-obesity effects remain unexamined. From the perspectives of translational medicine and clinical nutrition, the selection of specific lipids as delivery vehicles is of paramount importance. Although conventional fat replacers can reduce caloric intake, they frequently impede the absorption of lipophilic micronutrients [35]. Among digestible lipid carriers, while MCTs alone can solubilize carotenoids, they lack the LCFAs required to facilitate chylomicron assembly and subsequent lymphatic transport—the primary route for the efficient absorption of carotenoids [36]. Conversely, whereas LCTs can enhance carotenoid bioavailability, their excessive intake exacerbates postprandial lipemia and lipid accumulation. MLCTs, however, offer a dual advantage: their MCFA components undergo rapid β-oxidation and exhibit lower adipogenic potential, while their LCFA components facilitate the solubilization and lymphatic transport of carotenoids. Recent studies have demonstrated that structured MLCTs, inspired by human milk fat, serve as efficacious delivery systems that significantly enhance the digestibility and bioaccessibility of lutein compared with conventional lipids [37]. Therefore, investigating the synergistic effects of MCTs and MLCTs with carotenoids, along with their underlying mechanisms, is of profound scientific significance and practical value.

To determine how triglyceride structure affects the efficacy and safety of carotenoid supplements in obesity management, we used a high-fat diet (HFD)-induced obese C57BL/6J mouse model. We compared three lipid formulations, including MCTs, MLCTs, and their physical mixture (MCTs + LCTs), each uniformly supplemented with natural carotenoids (predominantly β-carotene and α-carotene). The experimental design employed isocaloric diets (45% fat), where the lipid source served as the sole variable. For comparison, a control group maintained on a low-fat diet was incorporated. During the ten-week intervention period, we tracked energy intake, body weight, and body composition, while also analyzing adipose tissue distribution, serum biochemical markers, liver function, and gut microbiota. This comprehensive approach was designed not only to compare efficacy but also to investigate the underlying mechanisms, particularly the role of gut microbiota, in the distinct responses elicited by these lipid-carotenoid combinations. Our findings provide key insights for designing combined formulations of structured lipids and fat-soluble bioactive compounds in precision nutrition strategies against obesity.

## 2. Materials and Methods

### 2.1. Materials

MCTs, MLCTs, and LCTs used in the experimental groups were prepared by Bunge Loders (Xiamen, China) Oils Technology Co., Ltd. (Xiamen, China). Natural carotenoids from red palm oil concentrate mixture were provided by PhytoGaia (Kuala Lumpur, Malaysia). The fatty acid methyl ester standards, α-carotene standards, and β-carotene standards were acquired from ANPEL Laboratory Technologies (Shanghai, China). Sinopharm Chemical Reagent Co., Ltd. (Shanghai, China) supplied all remaining reagents, all of which were analytical grade.

### 2.2. Methods

#### 2.2.1. Analysis of Carotenoid Content in Natural Carotenoid Complex

The natural carotenoids used in this experiment were analyzed for α-carotene, β-carotene, and total carotenoid content following the China National Standard for carotene determination in food (GB 5009.83–2016) [38]. Separation was achieved using reversed-phase chromatography, with quantification performed via the external standard method. An Agilent 1260 Infinity II HPLC system (Waldbronn, Germany) was used for the identification and quantification of total carotenoids, such as α-carotene and β-carotene. An Agilent C8 column (5 µm, 4.6 mm × 150 mm, Waldbronn, Germany) was used for separation. The mobile phase consisted of solution A (methanol/acetonitrile/water, 73.5:24.5:2, *v*/*v*) and solution B (methyl tert-butyl ether). The flow rate was maintained at 1.0 mL/min, with the column temperature set to 25 °C and detection monitored at 450 nm. All analyses were conducted in duplicate.

#### 2.2.2. Determination of Moisture Content in Lipid

A Mettler Toledo HE53/02 analyzer (Mettler-Toledo International Inc., Greifensee, Switzerland) was employed to measure moisture content in the oil samples. After turning on and equilibrating the analyzer for 30 min, 0.5 mL of MLCTs was placed in the center of the tray using a pipette, and the measurement was started. The analyses were performed in duplicate.

#### 2.2.3. Determination of Acid Value in Lipid

In line with the national food safety standard GB5009.229-2016 [39], the oil sample was dissolved in an organic solvent. Titration with a standardized sodium hydroxide solution was used to quantify free fatty acids. The endpoint was identified through the color change in the indicator, and the acid value was calculated using the volume of the consumed standard solution. The analyses were performed in duplicate.

#### 2.2.4. Determination of Peroxide Value in Lipid

The oil sample was dissolved in a chloroform-acetic acid solution following the national food safety standard GB5009.227-2016 [40]. Following the reaction between peroxides and potassium iodide to generate iodine, the liberated iodine was quantified by titration with a standardized sodium thiosulfate solution. The peroxide value was reported as the amount of active oxygen per kilogram of the sample, and the analyses were conducted in duplicate.

#### 2.2.5. Determination of Fatty Acid Composition (FAC) in Lipid

In accordance with the national food safety standard GB 5009.168–2016 [41], oil samples were transformed into fatty acid methyl esters (FAMEs) before analysis. Analysis was performed using an Agilent 7890B gas chromatograph (Agilent Co., Santa Clara, CA, USA) equipped with a flame ionization detector (FID) and an Agilent HP-88 capillary column (0.25 mm × 100 m × 0.20 μm). All samples were analyzed in duplicate.

#### 2.2.6. Diets

Jiangsu Xietong Pharmaceutical Bio-engineering Co., Ltd. (Nanjing, China) prepared the low-fat diet (maintenance food), 60% HFD, and 45% HFD used in this study. The feed formulation was designed according to the project’s experimental purpose, as detailed in Table 1 and Table 2. Carotenoids were dissolved in three types of oil vehicles, including MCTs, MLCTs, and MCTs + LCTs (a physical mixture of MCTs and LCTs). The FAC of the lipids is presented in Table 3 and representative gas chromatography (GC) chromatograms are provided in Supplementary Figure S1. Notably, the ratio of C8:0 to C10:0 in the MCTs was 1.43. Furthermore, the concentrations and high-performance liquid chromatography (HPLC) profiles of the natural carotenoids are detailed in Section 3.1. Experimental diets were prepared under low-temperature conditions (4 °C). Given the intrinsically lower caloric value of MCTs compared to LCTs, the theoretical energy density of the MCT-C diet was slightly lower. However, an ad libitum feeding model was employed to preserve the impact of the diet’s inherent energy composition on appetite and feeding behavior.

#### 2.2.7. Determination of Lipid Content in the Experimental Diets

Fat content in the experimental diets was determined following the Chinese national standard method GB/T 6433-2025 [42].

#### 2.2.8. Establishment of the Obesity Animal Model

Shanghai Lingchang Biotechnology Co., Ltd. (Shanghai, China) provided six-week-old male C57BL/6J mice (specific pathogen-free, SPF grade; initial body weight 22 ± 2 g). Throughout the study, mice were housed in an SPF facility maintained at 20–26 °C and 40–70% relative humidity. After seven days of acclimatization, animals were randomly allocated to either a control group (*n* = 15; low-fat diet) or a model group (*n* = 68; 60% HFD), as illustrated in the experimental design schematic. Sample sizes were determined based on previously published studies using identical diet-induced obese mouse models and similar outcome measures [43,44]. The primary outcome measure for sample size determination was body weight change during the dietary intervention. Simple random allocation was conducted manually without specialized statistical software. To minimize potential confounders, the order of treatments and measurements was randomized. Cage locations were randomized throughout the study period. Experimental procedures were performed by four trained operators following standardized protocols. Mice were weighed at least once per week. Upon observing that HFD-fed animals had gained significantly more weight than the control group after about three weeks (*p* < 0.05), the HFD-fed mice were ordered by body weight. The top two-thirds of mice with the highest body weight were selected as successful obesity models for subsequent nutritional intervention studies, while the bottom one-third (with lower weight gain) were excluded.

#### 2.2.9. Animals and Experimental Design

From the HFD-fed obese mice, three experimental groups were formed by random assignment (*n* = 15 per group): the MCT-C group (MCTs + 150 mg carotenoids/kg diet), the MLCT-C group (MLCTs + 150 mg carotenoids/kg diet), and the MCT+LCT-C group (physical mixture of MCTs and LCTs + 150 mg carotenoids/kg diet). The random assignment was performed manually using the same simple randomization method. All HFD groups received 45% of calories from fat, with fat sources being MCTs, MLCTs, or MCTs + LCTs, respectively (Table 1 and Table 2), compared to a control group consuming a low-fat purified diet. The ten-week study included weekly measurements of food intake (based on added, spilled, and remaining feed) and body weight to monitor changes. All animal procedures were performed in compliance with the guidelines of the Research Ethics Committee at Shanghai Jiao Tong University School of Public Health (Approval No. JUMC2024-262-A, approved on 12 December 2024).

#### 2.2.10. Body Composition Assessed via Low-Field Nuclear Magnetic Resonance Imaging

After the dietary intervention, the body weight of the mice was recorded, and body composition, including fat mass, lean mass, and total water content, was measured by an EchoMRI-100H (EchoMRI LLC, Houston, TX, USA). The lean mass-to-body weight ratio (lean mass coefficient), fat mass-to-body weight ratio (fat mass coefficient), and total water-to-body weight ratio (water coefficient) were subsequently calculated.

#### 2.2.11. Biochemical Index Measurement

Following the MRI examination, the mice underwent a 12 h fasting period before being euthanized under light anesthesia induced by an intraperitoneal injection of 1% pentobarbital sodium at a dose of 0.5 mL per 100 g body weight. Blood obtained by cardiac puncture was centrifuged (7000 rpm, ten minutes, 4 °C) to separate serum, which was then stored at −80 °C. Serum levels of triglycerides (TG), total cholesterol (TC), high-density lipoprotein cholesterol (HDL-C), low-density lipoprotein cholesterol (LDL-C), glucose, insulin, leptin, and non-esterified fatty acids (NEFA) were determined with specific assay kits. Kits for TG, TC, HDL-C, LDL-C, insulin, glucose, and leptin were sourced from ZCIBIO Technology Co., Ltd. (Shanghai, China); the NEFA kit came from FUJIFILM Wako Pure Chemical Corporation (Osaka, Japan). Hepatic function was assessed by quantifying ALT and AST activities using kits from Nanjing Jiancheng Bioengineering Institute (Nanjing, China).

#### 2.2.12. Fat Pad and Liver Weight

Fat pads, including brown adipose tissue (BAT), subcutaneous adipose tissue (SAT), and visceral adipose tissue (VAT), were removed, weighed, and then immersed in the dedicated fixative solution for adipose tissue (Wuhan Servicebio Technology Co., Ltd., Wuhan, China) for pathological examination and evaluation. The ratios of BAT to body weight (BAT coefficient), SAT to body weight (SAT coefficient), and VAT to body weight (VAT coefficient) were calculated. In the meantime, livers were taken out, weighed, and paraformaldehyde was added for further pathological examination and assessment. The liver-to-body weight ratio (liver coefficient) was additionally determined.

#### 2.2.13. Histologic Examination

Liver tissue processing began with cutting and embedding samples in Tissue-Tek directly onto the cryostat mold (CRYOSTAR NX50, Thermo Fisher Scientific China Co., Ltd., Shanghai, China). After air-drying for one hour, sections were rehydrated in distilled water (five minutes) and transferred to absolute propylene glycol (two minutes). Overnight staining with Oil Red O working solution was performed, followed by a one-minute immersion in 85% propylene glycol and a distilled water rinse. Sections then received a brief counter-stain with Harris hematoxylin (two to three seconds), dipped two to three times in 1% HCl alcohol, rinsed with water, and mounted using glycerol gelatin. Imaging was conducted with a NIKON ECLIPSE E100 microscope (Nikon Corporation, Tokyo, Japan).

Fat pads of mice, including BAT, SAT, and VAT, were collected for weight measurement and histopathological examination. Excessive lipid accumulation within adipose tissue is a hallmark characteristic of obesity. Lipid deposition was assessed by hematoxylin and eosin (H&E) staining, and the mean adipocyte area was calculated from measurements of individual adipocytes using image analysis software. This measurement of average cross-sectional area is a standardized histomorphometric approach widely utilized in previous metabolic studies to evaluate adipocyte hypertrophy [45]. For each sample, three randomly selected microscopic fields were examined.

#### 2.2.14. Metagenomic Sequencing of the Mouse Gut Microbiome

DNA extraction from mouse feces was carried out with the Magnetic Soil and Stool DNA Kit (TIANGEN, Beijing, China). Quality assessment involved 1% agarose gel electrophoresis for integrity and the Qubit dsDNA Assay Kit (Thermo Fisher Scientific, Waltham, MA, USA) for concentration. Qualified DNA samples were used for library construction. Libraries were prepared from 1 µg of DNA per sample employing the NEBNext^®^ Ultra™ DNA Library Prep Kit for Illumina (NEB, Ipswich, MA, USA). An Agilent 2100 Bioanalyzer (Agilent Technologies, Santa Clara, CA, USA) verified fragment size, while a Bio-Rad CFX96 real-time PCR system (Bio-Rad, Hercules, CA, USA) determined library concentration. Sequencing was conducted on an Illumina NovaSeq 6000 platform (Illumina, San Diego, CA, USA) with paired-end 150 bp reads.

Raw sequencing data were subjected to quality control using fastp (v.0.23.4). Reads were trimmed utilizing a sliding window of 6 bp, and truncation occurred when the average Phred quality score within the window fell below 20. Subsequently, reads shorter than 100 bp after trimming, or those identified as host contamination, were discarded. The high-quality reads were then de novo assembled using MEGAHIT (v.1.2.9), retaining only contigs with a length of ≥500 bp. Coding sequences (CDSs) were subsequently predicted from the assembled contigs employing MetaGeneMark (v.3.26). To construct a non-redundant gene catalog, the predicted gene sequences were dereplicated utilizing MMseqs2 (v.15-6f452). Taxonomic annotation was performed by aligning the non-redundant sequences against the microbial subset of the NCBI non-redundant database (NR_meta database) using DIAMOND, applying an E-value threshold of ≤10^−5^.

Principal coordinate analysis (PCoA) and non-metric multidimensional scaling (NMDS), based on Bray–Curtis dissimilarities, were employed as multivariate approaches to evaluate compositional differences among groups at both the taxonomic and functional levels. For the relative abundances of individual taxa or functional pathways, statistical comparisons were conducted using the Wilcoxon rank-sum test for two groups, and the Kruskal–Wallis test for multiple groups. Furthermore, the linear discriminant analysis effect size (LEfSe) method was utilized to identify taxa differentially enriched in specific groups. Significance thresholds were set at an alpha value of 0.05 for the non-parametric factorial Kruskal–Wallis rank-sum test, and a logarithmic LDA score threshold of >3.0 was applied to determine the discriminative features.

For the species and functional contribution analysis, the algorithm relied on the dual annotation of the non-redundant gene catalog (Unigenes) against both the NR_meta and KEGG databases. To determine the taxonomic contributors to a specific target functional pathway, the relative abundances of all Unigenes simultaneously assigned to that pathway and a specific species were aggregated, thereby calculating the relative contribution of each species to that function. Based on these calculated values, the top 20 representative species contributing most significantly to the abundance of the target pathway were extracted.

#### 2.2.15. Statistical Methods

Data processing and statistical analyses were performed with GraphPad Prism 9.0 (GraphPad Software, LLC, Boston, MA, USA) and SPSS 31.0 (IBM SPSS Statistics, IBM Corp., Armonk, NY, USA). Descriptive statistics are presented as mean ± standard deviation (SD) for normally distributed data, or median and interquartile range (IQR) for non-normally distributed data. Standard error of the mean (SEM) is used for error bars in all figures unless otherwise specified. Normality was assessed using the Shapiro–Wilk test. For normally distributed data, Bartlett’s test was used to evaluate the homogeneity of variance; for non-normally distributed data, the Brown-Forsythe test was used.

For data with normal distribution and equal variances, group differences among multiple groups were analyzed using ordinary one-way ANOVA, and Tukey’s post hoc test was used for multiple comparisons. For data with normal distribution but unequal variances, Brown-Forsythe and Welch ANOVA tests were applied, followed by Tamhane’s T2 test for multiple comparisons. For non-normally distributed data, the Kruskal–Wallis test was used for multiple group comparisons, and Dunn’s test was used for multiple comparisons. Statistical significance was assumed at the 95% level (*p* < 0.05).

Metagenomic data visualization included bar plots, heatmaps, and cluster analysis to illustrate community structure. Alpha diversity was assessed using the Observed species, Chao1, Shannon, and Simpson indices, with group differences analyzed via the Wilcoxon/Kruskal–Wallis test. Beta diversity was evaluated based on the Bray–Curtis distance matrix, employing PCoA/NMDS for dimensionality reduction, and ANOSIM/Adonis tests to assess between-group differences. Differential species and functions were identified using the Wilcoxon/Kruskal–Wallis test, STAMP, and LEfSe analysis. Functional annotation and species contribution analysis were conducted using the carbohydrate-active enzymes database (CAZy) and the Kyoto Encyclopedia of Genes and Genomes (KEGG) database as a reference. All analyses were conducted on the R platform (v.3.6.0) using packages such as vegan, ade4, and phyloseq.

## 3. Results and Discussion

### 3.1. Quantification of Carotenoids in the Natural Carotenoid Mixture and Formulated Diets

**Figure 1:**
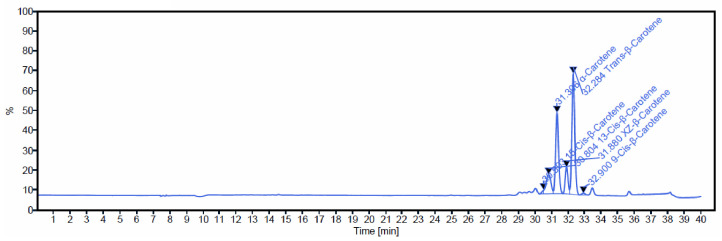
HPLC chromatogram of carotenoids in a natural palm mixed-carotenoid complex.

HPLC analysis confirmed a high concentration of carotenoids in the natural palm complex, with a total content of 347.5 ± 14.85 mg/g (Figure 1). The composition was characterized by 239.0 ± 9.90 mg/g of β-carotene and 108.5 ± 4.95 mg/g of α-carotene. These quantified values served as the basis for precisely formulating the experimental diets to ensure consistent carotenoid dosing across all intervention groups.

### 3.2. Physicochemical Characteristics of Experimental Oils

The physicochemical characteristics of the experimental oils (MCTs, MLCTs, and the MCT + LCT physical mixture) were analyzed, including acid value, peroxide value, moisture content, and fatty acid composition (FAC) (Table 3 and Table 4).

FAC analysis revealed that MCTs consisted almost entirely of MCFAs, predominantly octanoic acid (C8:0; 58.03%) and decanoic acid (C10:0; 40.45%). In contrast, both the structured MLCTs and the MCT + LCT physical mixture were predominantly composed of long-chain fatty acids (LCFAs), with nearly identical LCFA contents of 83.69% and 83.71%, and MCFA contents of 16.16% and 16.22%, respectively. Crucially, the detailed FAC analysis confirmed that the MCT + LCT physical mixture was successfully formulated to match the overall fatty acid profile (including the ratio of MCFAs to LCFAs and the proportions of individual fatty acids such as C18:1 and C18:2) of the structured MLCTs oil (Table 3). This design allowed for the specific investigation of the effects of triglyceride structure (randomized esterification in MLCTs vs. physical blend) independently of gross fatty acid composition.

### 3.3. Lipid Content of the Formulated Diets

**Figure 2:**
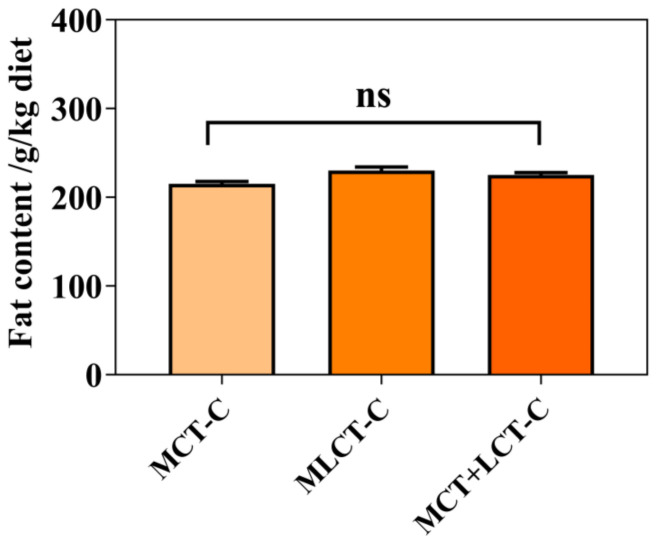
Lipid content of the experimental diets in the MCT-C, MLCT-C, and MCT+LCT-C groups. (ns: *p* > 0.05).

The analyzed lipid content of the experimental diets was comparable across all high-fat groups, with values of 215.0 ± 2.8, 230.0 ± 4.2, and 225.0 ± 2.8 g/kg for the MCT-C, MLCT-C, and MCT+LCT-C groups, respectively (Figure 2). Statistical analysis using one-way ANOVA showed no significant differences in dietary lipid levels across the three experimental groups (*p* > 0.05).

### 3.4. Validation of the Obesity Animal Model

**Figure 3:**
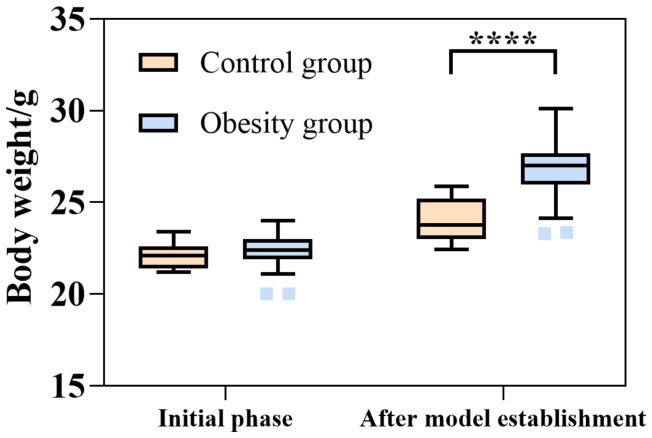
Body weight comparisons between experimental groups: Initial body weights of control (*n* = 15) and pre-obesity (*n* = 68) groups (*p* = 0.1510); Body weight after 3-week modeling period, showing significant divergence between the control group fed standard diet and the group fed an HFD (**** *p* < 0.0001).

As shown in Figure 3, C57BL/6J mice in both the control (*n* = 15) and pre-obesity (*n* = 68) groups started with comparable initial body weights (22.07 ± 0.67 g vs. 22.40 ± 0.83 g; *p* = 0.1510). After three weeks of dietary intervention, significant differences in body weight emerged among the groups. Mice fed a low-fat diet maintained a weight of 23.99 ± 1.18 g, whereas those on a 60% HFD developed significant obesity, reaching 26.72 ± 1.44 g (*p* < 0.0001), confirming the successful establishment of the diet-induced obesity (DIO) model.

To obtain a homogeneous obese cohort for the intervention study, the HFD-fed mice were ranked by body weight gain. The bottom tertile (one-third) of mice displaying the lowest weight gain, deemed obesity-resistant, were excluded. The remaining mice, representing the diet-induced obese population, were then randomly assigned to the experimental intervention groups [46].

### 3.5. Effects of Experimental Diets on Body Weight and Food Consumption of Obese Mice Induced by an HFD

**Figure 4:**
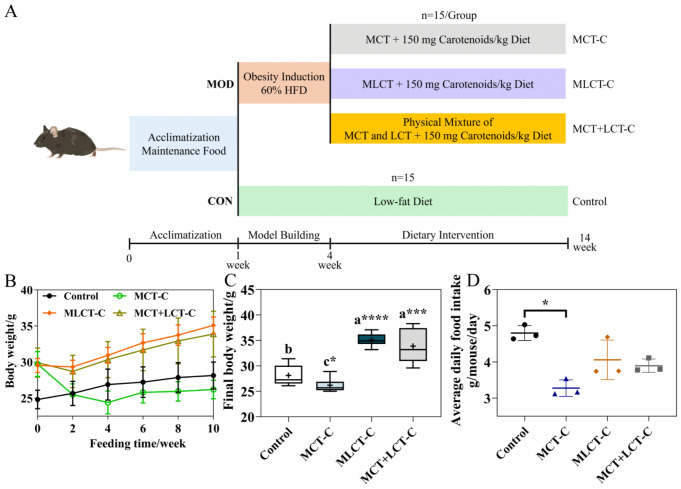
Effects of different dietary interventions on body weight and food intake in mice. (**A**) Experimental timeline and group allocation schematic. (**B**) Longitudinal body weight progression curves. (**C**) Terminal body weight measurements (*n* = 12 per group). (**D**) Average daily food intake. Data points in (**C**) marked with different lowercase letters (a–c) are statistically significantly different (*p* < 0.05). Asterisks denote significant differences compared to the control group (* *p* < 0.05, *** *p* < 0.001, **** *p* < 0.0001).

Weekly body weight measurements showed distinct changing patterns across all experimental groups (Figure 4B). Throughout the experimental period, the control group exhibited consistent weight gain. In contrast, the HFD groups exhibited an initial decline in body mass during the early intervention phase. Among the HFD groups, divergent patterns emerged thereafter. The body weight of the MLCT-C group remained stable in the first two weeks before increasing steadily until the end of the experiment. A similar trend was observed in the MCT+LCT-C group, which showed an initial decrease followed by a progressive weight increase. The MCT-C group displayed the most pronounced response: body weight consistently decreased until week four, surged markedly between weeks four and six, and then continued to increase at a slower rate until week ten. This initial weight reduction in HFD groups may reflect a transient period of dietary adaptation.

Final body weights of mice following the ten-week intervention are presented in Figure 4C. The MCT-C group exhibited the lowest final body weight (26.20 ± 1.28 g), a value significantly lower than that of the control group (28.14 ± 1.88 g) (*p* < 0.05). The MLCT-C and MCT+LCT-C groups exhibited the highest body weights, with no significant difference between them (*p* > 0.05). These terminal body weight data are consistent with the dynamic trends observed in the longitudinal growth curves (Figure 4B).

The HFD groups displayed significant differences in average daily food intake compared to the control group (Figure 4D). Consistent with expectations and prior findings [47], the control group demonstrated the highest daily intake (4.80 ± 0.20 g/mouse/day). This phenomenon is likely attributable to the lower energy density of the standard chow, necessitating greater consumption to meet energy demands. Although there were no significant differences among the HFD groups, the MCT-C group exhibited the lowest food intake (3.28 ± 0.23 g/mouse/day). This reduction may extend beyond palatability, as studies indicate that replacing LCTs with MCTs can enhance satiety signals, thereby curbing energy consumption [48,49]. This MCTs-induced satiety, combined with its slightly lower energy density, contributed to a reduction in total caloric intake in this group. This is the rationale for not employing isocaloric pair-feeding, which would have masked this critical physiological mechanism. No significant difference was observed between the MLCT-C and MCT+LCT-C groups (*p* > 0.05), suggesting that the structured lipid (MLCTs) and the physical mixture supported comparable energy acquisition. Collectively, these findings underscore the combined influence of dietary energy density and fatty acid composition in shaping feeding behavior.

### 3.6. Effects of Experimental Diets on Body Composition in Obese Mice Induced by an HFD

**Figure 5:**
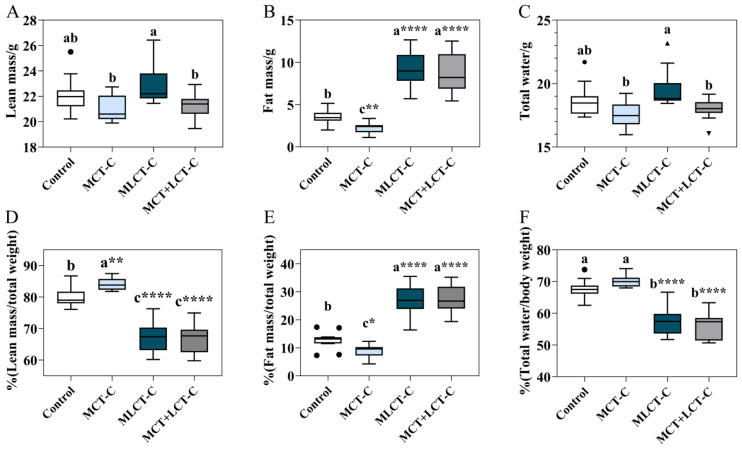
Modulation of body composition by different dietary lipids in obese mice (*n* = 12). (**A**) Lean mass, (**B**) Fat mass, (**C**) Total body water, (**D**) Lean mass coefficient, (**E**) Fat mass coefficient, and (**F**) Water coefficient. Lowercase letters (a–c) indicate significant intergroup differences (*p* < 0.05). Asterisks denote significant differences compared to the control group (* *p* < 0.05, ** *p* < 0.01, **** *p* < 0.0001).

Body composition analysis via quantitative MRI revealed distinct patterns of adipose tissue deposition, lean mass retention, and hydration status across experimental groups following the dietary intervention, with complete metrics documented in Figure 5.

Data in Figure 5A indicate that the MLCT-C group achieved the highest lean mass, significantly exceeding values recorded for both the MCT-C and MCT+LCT-C groups (*p* < 0.05). Figure 5B demonstrates that the MCT-C group had the lowest fat mass, even lower than the control group. By comparison, the MCT-C and MCT+LCT-C groups showed the lowest lean mass values, though no significant difference was observed between these two groups. Additionally, the total water content results mirrored the lean mass trends (Figure 5C). The MLCT-C group displayed the highest total water content, significantly surpassing the MCT-C and MCT+LCT-C groups (*p* < 0.05).

Figure 5D displays significant variations in lean mass coefficient across the groups. The MCT-C group had the highest lean mass coefficient, which was significantly higher than the control group (*p* < 0.05). Although the MLCT-C group exhibited the highest absolute lean mass (Figure 5A), this group exhibited a significantly lower lean mass coefficient than both the control and MCT-C groups (*p* < 0.05). This apparent discrepancy can be explained by the more substantial increase in overall body weight in the MLCT-C group, which proportionally reduced the lean mass to body weight ratio. No significant difference was observed between the MLCT-C and MCT+LCT-C groups, although a slightly higher trend was noted in the MLCT-C group.

A comparative analysis of lean mass coefficient (Figure 5D) and final body weight (Figure 4C) suggests a potential inverse relationship between these parameters in our study: mice with lower body weight tended to exhibit a higher proportion of lean mass, whereas increased body weight was associated with a reduced lean mass coefficient. This trend diverges in several aspects from the results documented by Graeme I. Lancaster et al. [50]. In that study, mice on an HFD gained significant body weight and fat mass without a significant change in absolute lean mass compared to controls, whereas mice on a normal diet gained weight primarily through an increase in lean mass. The distinct response observed in our study, particularly the inverse correlation between body weight and lean mass coefficient, may be attributed to differences in experimental conditions, such as the specific composition of the HFD or the duration of the intervention.

The experimental groups exhibited significant differences in fat mass coefficient (Figure 5E). Most notably, the MCT-C group exhibited the lowest fat mass coefficient, which was significantly lower than even that of the control group (*p* < 0.05), highlighting its potent effect on reducing adiposity. In contrast, the MLCT-C and MCT+LCT-C groups demonstrated substantially higher fat mass coefficients compared to both the control and MCT-C groups (*p* < 0.001). The MCT+LCT-C group displayed a slight upward trend that was not statistically significant. An inverse correlation was detected between fat mass coefficient and lean mass coefficient (Figure 5D), which suggested that alterations in body weight were mainly attributed to changes in fat mass. This strong positive correlation between body weight and adiposity is consistent with established findings in diet-induced obesity models. Supporting this relationship, Radlinger et al. (2023) reported that after a ten-week Western diet intervention, mice exhibited significantly higher body fat percentage and body weight compared to those fed a standard control diet [51].

Both the MLCT-C and MCT+LCT-C groups exhibited significantly increased body weight and adipose tissue accumulation, suggesting that the inclusion of LCFAs reduced the body-weight-lowering effect observed with MCT alone. In contrast, despite consuming an HFD, mice in the MCT-C group displayed significantly lower body weight and fat mass than the control group, suggesting reduced fat accumulation and enhanced energy utilization. Previous studies have shown that MCFAs are absorbed predominantly via the portal vein and rapidly transported to the liver, where they undergo preferential β-oxidation. This metabolic characteristic is associated with increased diet-induced thermogenesis and elevated energy expenditure, which may contribute to the reduced adiposity observed in the MCT-C group [20].

Figure 5F displays the water coefficient across experimental groups. This parameter showed trends comparable to the lean mass coefficient (Figure 5D) and was negatively correlated with body weight. The MCT-C group presented the highest water coefficient, with the control group ranking second. The MLCT-C and MCT+LCT-C groups showed significantly lower values relative to the control and MCT-C groups (*p* < 0.05). The MLCT-C group exhibited a slightly elevated water coefficient compared to the MCT+LCT-C group, although this difference did not reach statistical significance.

These findings collectively demonstrate that adiposity parameters are positively associated with body weight, whereas lean mass and hydration indices show an inverse relationship with body mass. This pattern differs from observations by Graeme I. Lancaster et al., who reported that mice on an HFD showed significant increases in body weight, fat mass, and fat mass percentage compared to a normal diet group. However, no significant intergroup differences were observed in either free water or total water content. This trend conflicts with the strong correlation between hydration level and lean mass identified in our current study [50].

### 3.7. Effects of Experimental Diets on Adipose Tissue and Fat Pad Coefficients in Obese Mice Induced by an HFD

**Figure 6:**
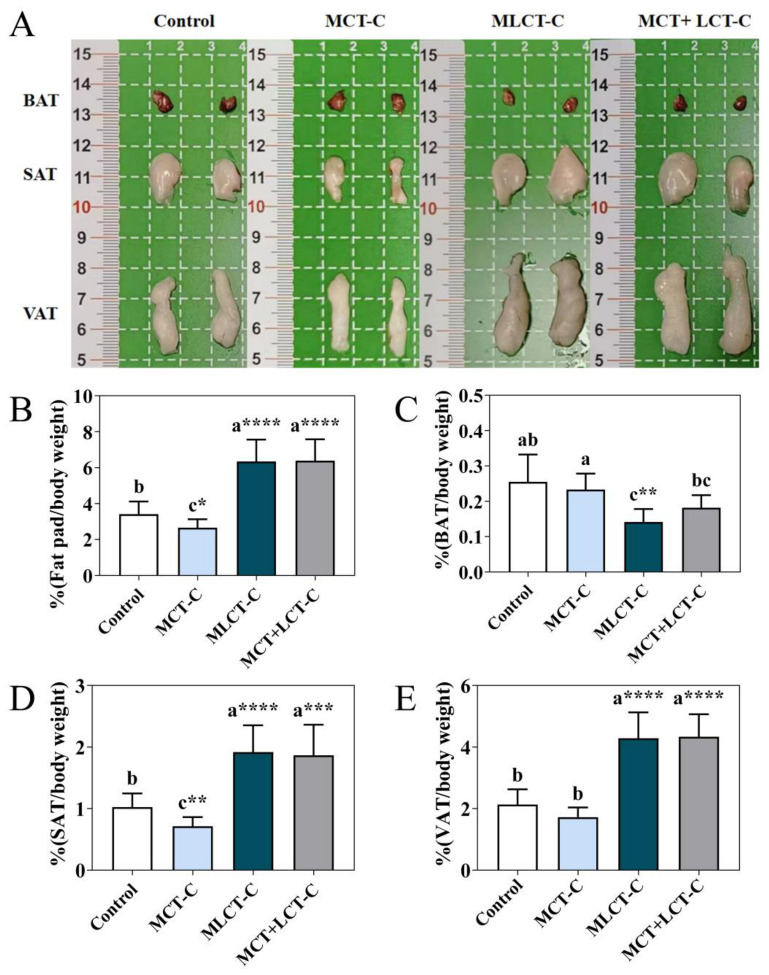
Adipose tissue mass and distribution in mice fed experimental diets. (**A**) Gross morphology of adipose depots. (**B**–**E**) Tissue mass normalized to body weight (coefficients) (*n* = 12 per group): (**B**) Total fat pad, (**C**) BAT, (**D**) SAT, and (**E**) VAT. Lowercase letters (a–c) in panels (**B**–**E**) indicate significant intergroup differences (*p* < 0.05). Asterisks denote significant differences relative to the control group (* *p* < 0.05, ** *p* < 0.01, *** *p* < 0.001, **** *p* < 0.0001).

White adipose tissue (WAT), the primary site of lipid storage, regulates immune responses through secretory mediators [52]. Following the dietary intervention, BAT, SAT, and VAT were harvested from the experimental mice for weight measurement and histological examination. The anatomical appearance of the adipose depots revealed distinct variations among the groups (Figure 6A). BAT was characterized by its crimson color and notably smaller volume compared to the white adipose depots. In terms of brown adipose pads, those from the control and MCT-C groups were visibly larger than the pads from the MLCT-C and MCT+LCT-C groups. Conversely, an opposite trend was observed for the white adipose pads (SAT and VAT). The MCT-C group possessed the smallest white adipose pads, which were even less substantial than those in the control group. In contrast, the MLCT-C and MCT+LCT-C groups exhibited markedly larger SAT and VAT depots compared to both the control and MCT-C groups. A subtle, non-significant trend indicated that the MCT+LCT-C group had marginally larger white adipose pads than the MLCT-C group. These morphological differences suggest that the dietary lipids differentially influenced the expansion of various adipose depots.

These morphological observations provide anatomical evidence that supports the quantitative data on body weight (Figure 4C) and fat mass coefficient (Figure 5E), collectively indicating a marked expansion of WAT alongside a reduction in brown adipose mass in obese mice.

As shown in Figure 6B, the fat pad coefficient (comprising BAT, SAT, and VAT) yielded results consistent with the body composition data in Figure 5E. Specifically, the MLCT-C and MCT+LCT-C groups presented significantly higher values compared with the control and MCT-C groups (*p* < 0.05), with the MCT+LCT-C group exhibiting a non-significant trend toward a higher coefficient than the MLCT-C group. Relative to the control group, the MCT-C group displayed a significantly reduced fat pad coefficient, representing the lowest value observed (*p* < 0.05). These quantitative findings were corroborated by the gross anatomical appearance of the fat pads shown in Figure 6A.

Figure 6C displays the BAT coefficient across the experimental groups. In contrast to the total fat pad coefficient (Figure 6B), the control and MCT-C groups exhibited the highest BAT coefficients. Specifically, the BAT coefficient in the control group was significantly higher than that in the MLCT-C group (*p* < 0.05), while the MCT-C group presented a markedly elevated BAT coefficient relative to both the MLCT-C and MCT+LCT-C groups (*p* < 0.05). A significantly reduced coefficient was observed in the MLCT-C group compared to the MCT+LCT-C group (*p* < 0.05). These measured values correspond well with the gross BAT morphology illustrated in Figure 6A.

Figure 6D and Figure 6E present the SAT and VAT coefficients across experimental groups, respectively. The trends of both ratios were consistent with the fat pad coefficient presented in Figure 6B. The MCT-C group exhibited the lowest SAT and VAT coefficients, with the SAT coefficient being significantly lower than that of the control group (*p* < 0.05). Conversely, both the MLCT-C and MCT+LCT-C groups exhibited significantly higher SAT and VAT coefficients than both the control and MCT-C groups (*p* < 0.05). Notably, the MCT+LCT-C group showed a trend toward a higher VAT coefficient relative to the MLCT-C group. These quantitative findings correlated well with the anatomical appearance of WAT observed in the mice (Figure 6A).

The analysis of experimental results suggests a consistent association between body weight and adipose tissue characteristics in obese mice. Specifically, higher body weight coincided with larger WAT volumes but smaller BAT size and coefficients. Conversely, lower body weight was associated with reduced WAT volumes but notably larger BAT measurements. The differential remodeling of white and brown adipose tissue observed in our study is consistent with findings from other nutritional studies. For instance, Wang et al. (2023) reported that Panax notoginseng saponins (PNS) significantly reduced both VAT and SAT volumes in obese mice, while slightly increasing the BAT coefficient [47]. This pattern of changes in specific fat depots is consistent with the distinct effects induced by the different lipid-carotenoid formulations in our experiment.

### 3.8. Effect of Different Diets on Adipocyte Size and Adipose Tissue Morphology in Obese Mice Induced by an HFD

WAT represents the major adipose tissue for fat storage [52]. Extensive research has demonstrated that rodents subjected to HFD for periods exceeding four weeks exhibit significant alterations in adipose tissue morphology and function. Within WAT depots, these dietary treatments promote adipocyte hypertrophy (enlargement) and hyperplasia (increased cell count). Furthermore, HFD alters the secretion of key adipokines like leptin and adiponectin, which play critical roles in energy homeostasis and metabolic regulation [53]. These observations highlight the dynamic nature of WAT in responding to nutritional challenges and its central role in the pathogenesis of diet-induced obesity and associated metabolic disorders.

**Figure 7:**
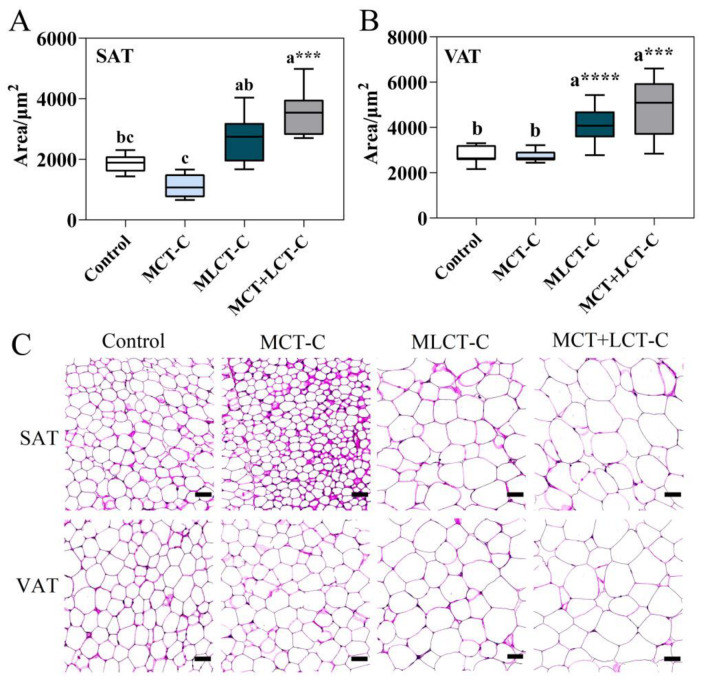
Adipocyte size and morphology in C57BL/6J mice under different diets (*n* = 4 per group). (**A**) Adipocyte size of SAT; (**B**) Adipocyte size of VAT; (**C**) Representative adipose tissue morphology (SAT, top; VAT, bottom) across dietary groups. Lowercase letters (a–c) in panels (**A**,**B**) indicate significant intergroup differences (*p* < 0.05). Asterisks denote statistical significance relative to the control group (*** *p* < 0.001, **** *p* < 0.0001). The scale bar in (**C**) represents 50 µm.

The effects of different oil types combined with carotenoids on adipocyte area in SAT and VAT of HFD-fed obese mice are shown in Figure 7A and Figure 7B, respectively. In the SAT, adipocytes were the smallest in the MCT-C group, with sizes smaller than those in the LFD control group, while both the MLCT-C and MCT+LCT-C groups exhibited hypertrophy. Comparison between the two groups revealed that the adipocyte area in the MCT+LCT-C group was larger than that in the MLCT-C group, though the difference was not statistically significant (*p* > 0.05). VAT exhibited a corresponding pattern. The LFD control and MCT-C groups had similarly small adipocytes. Both the MLCT-C and MCT+LCT-C groups showed marked hypertrophy compared to the other groups. The MCT+LCT-C group exhibited larger adipocytes compared to the MLCT-C group, with no significant difference observed between the two groups (*p* > 0.05), which was consistent with the results of the SAT adipocyte area and the changing trend of VAT coefficient in Figure 6E. These results suggest that MLCTs and MCTs + LCTs exert distinct impacts on obesity. This differential adipocyte hypertrophy is fundamentally attributed to distinct intestinal digestion kinetics. As noted by Cheng et al., compared to a simple physical mixture, MLCTs alter the steric hindrance and specific hydrolysis rate of pancreatic lipase. In the physical mixture group, LCTs are independently and rapidly hydrolyzed and re-esterified into chylomicrons, leading to a massive and rapid postprandial influx of long-chain fatty acids into WAT. In contrast, the unique molecular structure of MLCTs reduces the absorption rate of LCTs. This steady and controlled digestion kinetics prevents acute postprandial lipid absorption, thereby mitigating the pressure of excessive lipid accumulation on individual adipocytes at the source [17].

Representative photomicrographs of H&E-stained SAT and VAT from each experimental group are displayed in Figure 7C. Morphological features matched the quantitative data for adipocyte area presented in Figure 7A,B. Specifically, the micrographs visually confirm the smallest adipocyte size in the MCT-C group for both SAT and VAT. Hypertrophic adipocytes were observed in the MLCT-C and MCT+LCT-C groups, with larger cell size in the MCT+LCT-C group relative to the MLCT-C group.

The consistency observed across adipocyte morphometry (Figure 7A,B), adipose tissue morphology (Figure 7C), anatomical appearance, and fat pad coefficients (Figure 6A,B) demonstrates a coherent pattern of adipose remodeling. Furthermore, these adipose-specific findings align with the higher-order physiological outcomes of final body weight (Figure 4C) and whole-body fat mass coefficient (Figure 5E), collectively reflecting the systemic metabolic outcomes induced by distinct dietary treatments.

The above experimental results demonstrate that, under conditions of equal carotenoid content, the type of dietary lipid significantly modulated body weight and adiposity in HFD-induced obese mice. Mice receiving the MCT-C diet showed significantly lower final body weight and fat mass than the control group. In contrast, the MLCT-C and MCT+LCT-C groups reached higher body weight and adiposity. Histological examination of WAT verified these phenotypic variations at the cellular level. Notably, although the MLCT-C and MCT+LCT-C groups showed comparable overall body weight and fat mass, the MCT+LCT-C group exhibited depot-specific hypertrophy, with larger adipocytes in both VAT and SAT compared to the MLCT-C group.

### 3.9. Effects of Experimental Diets on Liver Morphology, Weight, and Function in Obese Mice Induced by an HFD

**Figure 8:**
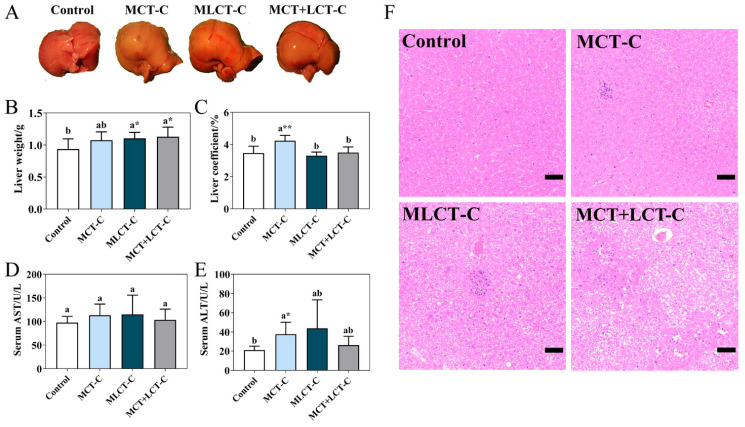
Hepatic evaluation in C57BL/6J mice under different dietary regimens. (**A**) Gross liver morphology, (**B**) absolute liver weight (*n* = 12 per group), (**C**) liver coefficient (*n* = 12 per group), (**D**) serum AST levels (*n* = 9 per group), (**E**) serum ALT levels (*n* = 9 per group), and (**F**) representative H&E-stained liver sections (20× magnification). Lowercase letters (a and b) in panels (**B**–**E**) indicate significant intergroup differences (*p* < 0.05). Asterisks denote statistical significance relative to the control group (* *p* < 0.05, ** *p* < 0.01). The scale bar in panel (**F**) corresponds to 50 µm.

Figure 8A presents the gross hepatic morphology observed in each experimental group. Macroscopic examination revealed that livers in the control group were slightly smaller than those in the HFD groups, with the HFD groups showing moderate hepatic enlargement. Notably, the livers of the MCT-C group exhibited a distinct granular appearance on the surface, characteristic of fatty liver pathology. In contrast, livers in all other groups maintained smooth surfaces without such morphological alterations.

As illustrated in Figure 8B, liver weights varied across groups. The MCT-C group displayed elevated liver weight relative to the control group, and this elevation was significant in both the MCT+LCT-C and MLCT-C groups (*p* < 0.05). No statistically significant differences were detected among the three high-fat diet groups. These quantitative findings are consistent with the gross morphological observations shown in Figure 8A.

As depicted in Figure 8C, the MCT-C group exhibited the highest liver coefficient, which was significantly higher compared with the control group and the other high-fat groups (*p* < 0.01). This pattern may be attributable to the liver damage visualized in Figure 8A. In contrast, no significant differences were observed between the other high-fat groups (MLCT-C and MCT+LCT-C) and the control group (*p* > 0.05).

Prior studies have reported both consistent and conflicting results in relation to our findings. Martin et al. found that increasing MCT content in the diet of mice fed MCTs combined with corn oil significantly attenuated liver pathological alterations [54]. Nevertheless, Janaina et al. reported that mice administered MCTs and fructose exhibited obvious hepatocellular steatosis and hepatic inflammation [55]. Chamma also noted that while supplementing an HFD with MCTs decreased fat accumulation and insulin resistance in mice, animals fed a very high MCT diet showed changes in hepatic lipid accumulation and metabolism, implying that long-term consumption of high-dose MCTs might be detrimental [56]. These experimental findings may suggest that while MCTs demonstrate potential to ameliorate hepatic pathological alterations, a higher dosage of MCTs might exert paradoxical effects, inducing hepatic cellular steatosis and other pathological changes.

Hepatic function was assessed by measuring serum AST and ALT levels in blood samples collected from each group (Figure 8D,E). MCT-C, MLCT-C and MCT+LCT-C groups showed a slight elevation in AST levels relative to the control group, but none of these differences reached statistical significance (*p* > 0.05). ALT was significantly elevated in the MCT-C group compared to the control group (*p* < 0.05). The MLCT-C and MCT+LCT-C groups showed mild ALT increases compared to the control group, that were not statistically significant (*p* > 0.05).

Figure 8F displays representative liver histopathological sections. Liver tissue from the control group displayed normal architecture without any notable pathological alterations. In the HFD groups, sporadic lymphocytic infiltration foci were occasionally detected. Notably, sporadic foci of hepatocyte necrosis were evident in the MCT+LCT-C group, but not in other groups. Varying degrees of hepatic congestion were present across all groups, being more noticeable yet non-severe in the MCT+LCT-C group. All groups exhibited mild focal vacuolar degeneration within hepatocytes. The observed hepatocyte necrosis and more pronounced congestion in the MCT+LCT-C group align with the notion that the hepatic impact of lipids is formulation-dependent. This observation agrees with earlier reports that high dietary MCT content can trigger hepatic abnormalities. In contrast, studies on structured lipids like MLCTs have demonstrated superior hepatic outcomes. Lee et al. reported a substantially smaller hepatic lipid deposition area in MLCT-fed mice compared to those fed a physical MCTs + LCTs mixture [43], suggesting that MLCTs’ unique molecular structure underlies its ameliorative effect. Supporting this, Zhou et al. found that increasing MCFA content in MLCTs led to a dose-dependent reduction in hepatic lipid accumulation [57], suggesting that MCFAs serves as the key component mediating the beneficial effects of MLCTs.

Unlike LCFAs that rely on lymphatic chylomicron transport, MCFAs derived from MLCT hydrolysis are directly absorbed into the liver via the portal vein, reducing their initial deposition in adipose tissue [58,59]. Importantly, MCFAs in hepatocytes bypass the carnitine shuttle to directly enter mitochondria, thereby driving β-oxidation [60]. This enhanced β-oxidation activates nuclear receptors like PPAR-α, which upregulate downstream lipolytic enzymes. Simultaneously, the resulting systemic metabolites facilitate cross-organ crosstalk via the circulation, promoting the thermogenic activation of BAT observed in this study [61]. Taken together with the phenotypic data, this metabolic axis provides the physiological basis for the synergistic metabolic regulatory effects of MLCTs combined with carotenoids.

### 3.10. Effect of Various Diets on Serum Biochemical Markers of Obese Mice Induced by an HFD

**Figure 9:**
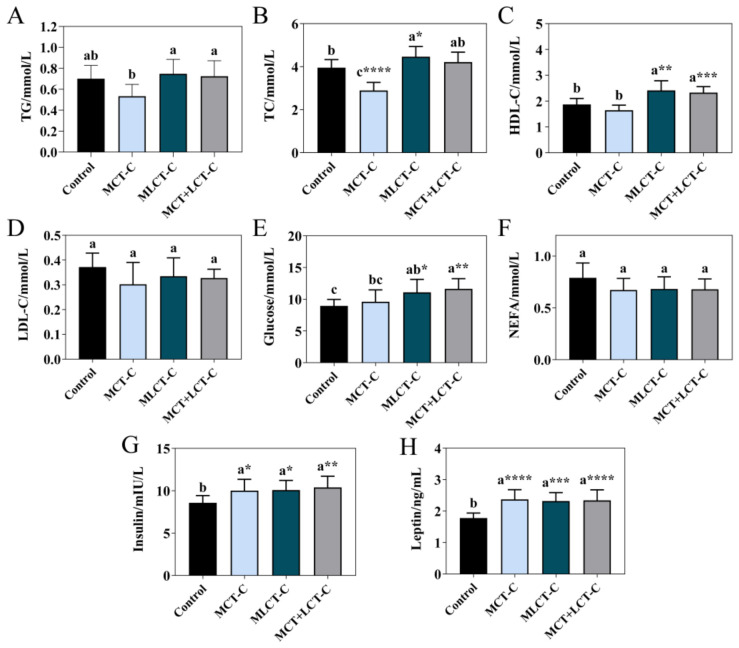
Effects of different diets on serum profiles in C57BL/6J mice (*n* = 11 per group). (**A**) TG, (**B**) TC, (**C**) HDL-C, (**D**) LDL-C, (**E**) glucose, (**F**) NEFA (*n* = 9 per group), (**G**) insulin, and (**H**) leptin. Lowercase letters (a–c) in panels (**A**–**H**) indicate significant intergroup differences (*p* < 0.05). Asterisks denote statistical significance relative to the control group (* *p* < 0.05, ** *p* < 0.01, *** *p* < 0.001, **** *p* < 0.0001).

Obesity is frequently associated with disturbances in lipid metabolism, with the most immediate and measurable manifestation being abnormal changes in blood lipid parameters [27]. Figure 9A illustrates the impact of various diets on serum triglyceride levels in high-fat-induced obese mice. The results demonstrated that the MCT-C group presented lower triglyceride levels relative to the control group. Nevertheless, no statistically significant differences in triglyceride levels were found between the MLCT-C and MCT+LCT-C groups relative to the control group (*p* > 0.05). Lee et al. observed that MLCT groups exhibited marginally lower serum TG levels than physically mixed groups under both low-fat and HFD, though these differences were not statistically significant (*p* > 0.05). These findings suggest that, relative to a physical MCTs + LCTs mixture, MLCTs exert only modest effects on serum TG levels.

Figure 9B presents the effects of various diets on TC levels in high-fat-induced obese mice. The MCT-C group exhibited the lowest TC levels (*p* < 0.0001), which differed significantly from those of the control group. The MLCT-C group displayed significantly higher TC levels than the control group (*p* < 0.05). In contrast, the MCT+LCT-C group did not differ significantly from the control group (*p* > 0.05). A direct comparison between the MLCT-C and MCT+LCT-C groups also revealed no statistically significant difference (*p* > 0.05). Hu et al. revealed that serum TC levels in the MLCT group were 16% lower compared to the high-fat group [26]. Yuan et al. found that although serum TC levels in the MLCT group were considerably lower than in the high-fat group, there was no statistically significant difference between the groups in comparison to the control group [62].

Figure 9C presents serum HDL-C levels in C57BL/6J mice across dietary treatments. Compared to the control group, HDL-C was significantly higher in both the MLCT-C (*p* < 0.01) and MCT+LCT-C groups (*p* < 0.001). HDL-C concentrations did not differ significantly between these two intervention groups (*p* > 0.05). In contrast, the MCT-C group exhibited the lowest HDL-C level, which was significantly lower than both the MLCT-C and MCT+LCT-C groups (*p* < 0.001). Zhou et al. found that HDL-C levels were significantly higher in MLCT groups composed of medium and high levels of MCFAs compared to the blank and obese controls. Conversely, the MLCT group with a low concentration of MCFAs, although higher than the controls, did not show a statistically significant difference. A positive association between dietary MCFAs from MLCTs and elevated HDL-C concentrations is suggested by these data [57].

Figure 9D illustrates the impacts of distinct dietary patterns on LDL-C levels in HFD-induced obese mice. Analysis of the data revealed generally comparable LDL-C levels across all experimental groups. Compared with the control group, the three HFD groups presented decreased LDL-C levels, yet none of the intergroup differences achieved statistical significance (*p* > 0.05). Yuan et al. found that the MLCT and physical mixture groups did not differ significantly from one another [62]. Other observations also documented that neither MLCTs nor rice oil (RO) significantly enhanced plasma HDL-C and LDL-C levels, which were adversely impacted by the HFD [27].

Figure 9E illustrates the effects of different dietary regimens on serum glucose levels in HFD-induced obese mice. Compared to the control group, both the MCT+LCT-C and MLCT-C groups showed significantly elevated serum glucose levels (*p* < 0.01 and *p* < 0.05, respectively). But no significant difference was found between these two groups (*p* > 0.05). The MCT-C group did not differ significantly from the control group (*p* > 0.05). These results indicate that both MLCTs and the physical mixture of MCTs and LCTs, in the presence of carotenoids, similarly affect serum glucose regulation, exerting a more pronounced hyperglycemic effect compared to MCTs with carotenoids alone.

Nonesterified fatty acids (NEFA), also referred to as free fatty acids, serve as key metabolic substrates and signaling molecules primarily generated through lipolysis in adipose tissue. Their levels in the circulation are closely tied to the regulation of lipid and glucose metabolism, as well as insulin sensitivity. Elevated circulating NEFA concentrations characterize various metabolic disorders such as obesity, type 2 diabetes, and insulin resistance, and are widely regarded as an important predictor of cardiovascular disease onset. As shown in Figure 9F, although no statistically significant differences were observed among the three HFD groups themselves, the MCT-C, MLCT-C, and MCT+LCT-C groups all exhibited lower mean serum NEFA levels compared to the control group, suggesting a consistent but non-significant trend of reduction induced by these dietary interventions in HFD-induced obese mice (*p* > 0.05).

The impact of different diets on serum insulin levels in HFD-induced obese C57BL/6J mice is depicted in Figure 9G. The data indicate no significant differences in serum insulin levels among the MCT-C, MLCT-C, and MCT+LCT-C groups (*p* > 0.05), as indicated by their shared statistical annotation. Serum insulin levels were significantly elevated in all three HFD-fed groups relative to the control group (*p* < 0.05). Relative to the control group, serum insulin levels rose by approximately 21.3% in the MCT+LCT-C group, 17.6% in the MLCT-C group, and 16.6% in the MCT-C group. This pattern of hyperinsulinemia is a well-established hallmark of HFD feeding and is consistent with the development of insulin resistance.

Figure 9H illustrates the impacts of distinct dietary patterns on serum leptin levels in HFD-induced obese mice. Experimental results demonstrate that all three HFD treatment groups exhibited significantly elevated serum leptin levels relative to the control group (*p* < 0.001). Although their mean values differed somewhat, no statistically significant differences were detected among these groups (*p* > 0.85). These results indicate that HFD consumption consistently and substantially elevates serum leptin levels, regardless of the specific fatty acid profile of the carotenoid-supplemented oils.

### 3.11. Analysis of Metagenomic Sequencing Results

#### 3.11.1. Influence of Various Structured Lipids on Murine Gut Microbiota Composition and Structure

**Figure 10:**
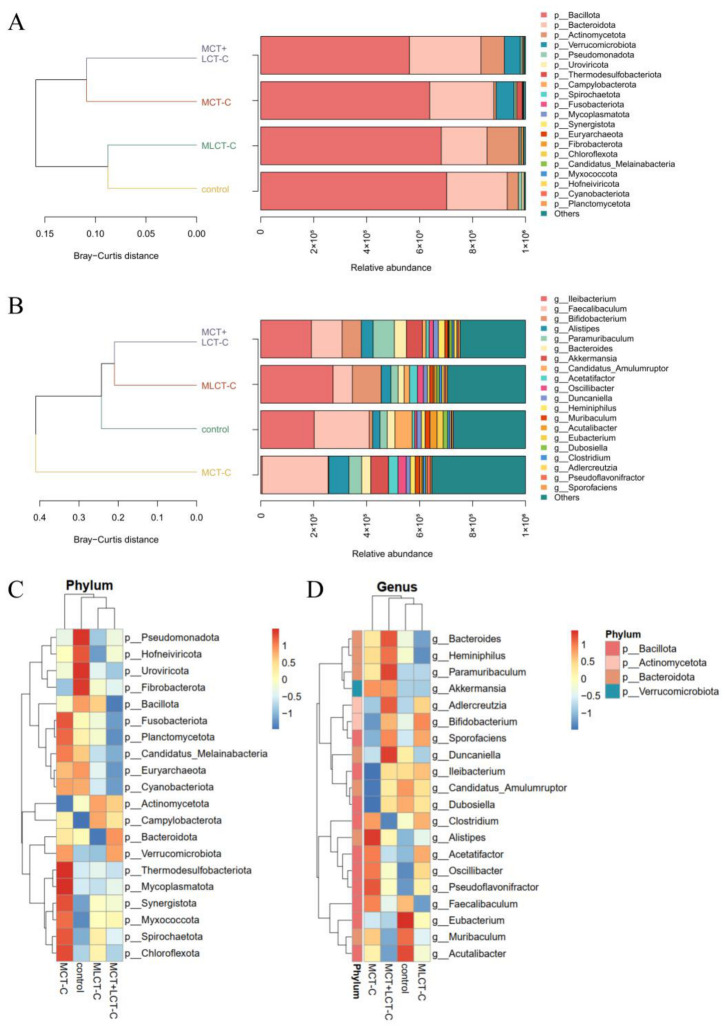
Analysis of the community structure and composition of gut microbiota in mice: (**A**) Community composition and cluster analysis at the phylum level; (**B**) Community composition and cluster analysis at the genus level; (**C**) Species abundance heatmap at the phylum level; (**D**) Species abundance heatmap at the genus level.

As shown in Figure 10A, the fecal microbiota at the phylum level primarily consisted of *Bacillota*, *Bacteroidota*, *Actinomycetota*, *Verrucomicrobiota*, and *Pseudomonadota*, along with a small proportion of unclassified bacteria. *Bacillota* and *Bacteroidota* dominated the gut microbiota in every experimental group, with combined relative abundances of 93.13% (Control), 85.48% (MLCT-C), 88.00% (MCT-C), and 83.20% (MCT+LCT-C). In the colonic environment, most *Bacillota* are butyrate-producing bacteria that generate short-chain fatty acids such as butyrate through carbohydrate fermentation, contributing to both colonic integrity and host energy homeostasis [63,64]. *Bacteroidota*, on the other hand, secrete various carbohydrate-active enzymes to degrade indigestible polysaccharides and produce short-chain fatty acids, playing a crucial role in maintaining intestinal energy balance [65].

Compared to the control group, the abundances of both *Bacillota* and *Bacteroidota* decreased in the MLCT-C group, but the decrease in *Bacteroidota* was more pronounced, leading to an increased *Bacillota*/*Bacteroidota* ratio (F/B ratio). In contrast, the MCT-C and MCT+LCT-C groups showed relatively lower F/B ratios. Previous studies have indicated that a higher F/B ratio is often associated with HFDs, weight gain, and obesity [57,63,66]. Nevertheless, some studies suggest that the F/B ratio may be decreased in obese individuals [67] and propose that this is related to alterations in lipid metabolite profiles, such as the metabolic regulation of triglycerides, phosphatidylcholines, and short-chain fatty acids [68]. These discrepancies may stem from differences in dietary composition, energy density, animal species, and duration of intervention.

The relative abundance of *Actinomycetota* increased in the MCT+LCT-C and MLCT-C groups but decreased in the MCT-C group, a trend largely driven by changes in *Bifidobacterium*. Furthermore, as shown in Figure 10C, the abundance of *Pseudomonadota* was the highest in the control group, followed by the MCT-C and MCT+LCT-C groups, while the MLCT-C group exhibited the lowest level. Previous studies have indicated that an increase in *Pseudomonadota* often reflects gut microbiota dysbiosis and serves as a key microbial marker of metabolic disorders [57]. Its excessive enrichment is closely associated with elevated endotoxin levels, intestinal barrier damage, and systemic low-grade inflammation, thereby promoting lipid metabolism disorders and weight gain [69,70]. Therefore, the significant reduction in *Pseudomonadota* abundance observed in the MLCT-C group (*p* < 0.001) may suggest a potential role in maintaining intestinal homeostasis and reducing inflammation risk.

Bray–Curtis cluster analysis showed that the phylum-level gut microbiota structure of the MLCT-C group was the closest to that of the control group. It can be seen from the phylum-level analysis that the MLCT-C group performed better in maintaining the proportion of major intestinal flora, increasing the abundance of *Actinomycetota*, and reducing the abnormal enrichment of *Pseudomonadota*, suggesting that phylum-level distribution of dominant gut bacteria in the MLCT-C group more closely resembled that of the control group.

To systematically identify the specific bacterial taxa driving the compositional shifts among the groups, LEfSe analysis was performed. Figure 10B reveals marked differences in fecal microbiota composition at the genus level across experimental groups based on the key biomarkers extracted from this analysis. Compared to the control group, the reduction in *Bacillota* in the MLCT-C group was primarily attributed to the LEfSe-identified depletion of *Faecalibaculum*. And the increased levels of *Faecalibaculum* have often correlated with insulin resistance, obesity, and gut microbiota dysbiosis [63,71].

As depicted in Figure 10D, the relative abundance of the phylum *Verrucomicrobiota* significantly increased in both the MCT-C and MCT+LCT-C groups, a shift primarily driven by the enrichment of the genus *Akkermansia*. As a mucin-degrading probiotic, *Akkermansia* is recognized as a crucial commensal bacterium for maintaining metabolic homeostasis; its abundance is negatively correlated with body fat mass and can counteract HFD-induced obesity [64,67]. Furthermore, it produces short-chain fatty acids (SCFAs) such as acetate to fortify the intestinal mucosal barrier and reduce permeability. This fortification prevents the translocation of gut-derived endotoxins into the systemic circulation, thereby alleviating metabolic disorders induced by a high-fat diet [72,73].

In contrast, LEfSe analysis highlighted *Bifidobacterium* as a significant core genus driving the increased relative abundance of *Actinomycetota* in the MCT+LCT-C and MLCT-C groups, but its decrease in the MCT-C group. As a representative probiotic genus, *Bifidobacterium* abundance closely reflects host metabolic health. It ferments dietary carbohydrates to produce lactic acid and acetic acid, thereby reinforcing gut barrier integrity, mitigating metabolic endotoxemia, influencing energy metabolic pathways, and suppressing inflammatory responses [66]. Meanwhile, it can also maintain intestinal homeostasis and lipid balance by competitively inhibiting potential pathogenic bacteria such as *Enterobacter* and *Enterococcus* and promoting bile acid excretion. Studies have shown that the abundance of *Bifidobacterium* is negatively correlated with BMI, and its reduction is often associated with metabolic disorders such as obesity and type 2 diabetes [63,73]. The significant increase in *Bifidobacterium* observed in the MCT+LCT-C and MLCT-C groups in the present study (*p* < 0.01) suggests that such interventions may shape a potential beneficial gut microbiota structure, but their specific functions may be subject to complex regulation by strain specificity and the overall gut microbiota environment.

Bray–Curtis cluster analysis showed that the genus-level gut microbiota structure of the MLCT-C group was the closest to that of the MCT+LCT-C group. Lipids with different structures may affect host metabolism by regulating the abundance of specific microbiota. The increase in *Bifidobacterium* after MLCT-C intervention provides potential microbiological perspectives for explaining how dietary strategies modulate obesity and metabolic disorders.

#### 3.11.2. Gut Microbiota Diversity and Functional Pathway Analysis in Mice

**Figure 11:**
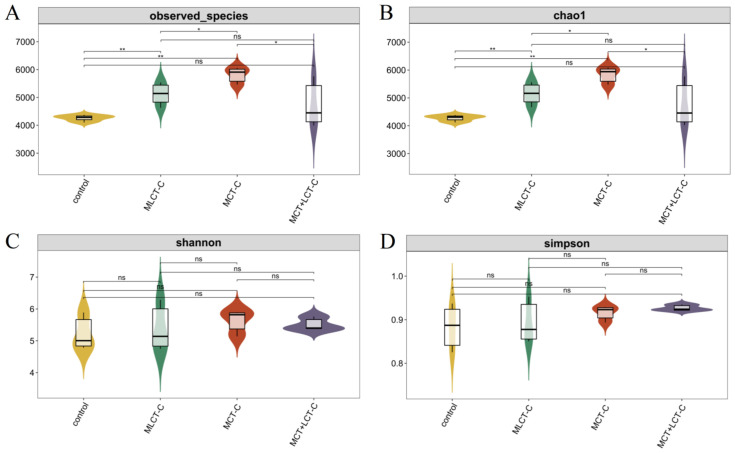
Alpha diversity analysis of the mouse gut microbiota: (**A**) Observed species index analysis; (**B**) Chao1 index analysis; (**C**) Shannon index analysis; (**D**) Simpson index analysis. Asterisks in (**A**,**B**) denote statistical significance between groups (* *p* < 0.05, ** *p* < 0.01), and ns in (**A**–**D**) indicates not significant.

Alpha diversity is a comprehensive indicator for evaluating the richness and diversity of gut microbiota in samples, with commonly used index standards including Observed species, Shannon, Simpson, and Chao1. Chao1 and Observed species primarily assess community richness, whereas the Shannon index and Simpson index indicate community diversity. Figure 11 demonstrates that the MCT-C and MLCT-C groups had significantly higher Observed species and Chao1 values than the control group (*p* < 0.05), indicating increased species richness following these treatments. This may be attributed to the increased relative abundance of specific taxa that can effectively utilize high-fat components in the diet and their metabolites. Species richness in the MCT+LCT-C group was comparable to that observed in the control group, with no significant difference detected (*p* > 0.05), indicating that this intervention was less effective than the other two schemes in improving gut microbiota richness in obese mice fed an HFD. In terms of community diversity, no significant differences in either the Shannon or Simpson index were detected between any of the treatment groups and the control group, indicating that the relevant interventions had little impact on community diversity.

**Figure 12:**
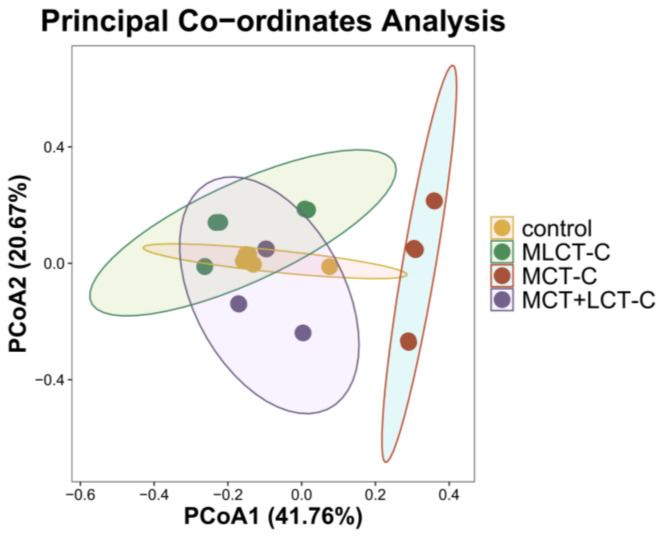
PCoA of beta diversity in the mouse gut microbiota.

Beta diversity analysis was employed to assess differences in microbial community structure among samples. As shown in Figure 12, in the PCoA at the OTU level, the two extracted principal components accounted for 62.43% of the total variance (PCoA1: 41.76% and PCoA2: 20.67%). Among the four groups, the MCT-C group was clearly separated from the other three groups, whereas the control, MLCT-C, and MCT+LCT-C groups partially overlapped in ordination space, indicating milder shifts in community structure for the latter two interventions.

As shown in Table 5, the trend revealed by PCoA was corroborated by Adonis analysis. Relative to the control group, as shown in the table, the MCT-C group had an R^2^ of approximately 0.555 with a significant *p*-value of 0.001, and the highest explanatory power. This indicates that MCT-C intervention exerted the most significant impact on the gut microbiota community structure of mice, which was consistent with the results of PCoA. In contrast, the other intervention groups had smaller R^2^ values and thus a lower portion of variance explained by treatment, indicating that MLCT-C and MCT+LCT-C induced more moderate, though still significant, divergence in community composition from the control group. This finding further indicated that, in contrast to the pronounced regulatory effect exerted by MCT-C, MLCT-C and MCT+LCT-C interventions induced milder alterations in the gut microbial community structure under an HFD background, suggesting their potential advantages in preserving the relative stability of the gut microbiota [23,74]. Such drastic alterations triggered by pure medium-chain triglycerides have been documented previously. For instance, Rial et al. noted that high-dose MCTs, through the rapid generation of short-chain fatty acids or ketone bodies during metabolism, can swiftly alter the intestinal microenvironmental pH, thereby affecting the resident microbiota [11,75]. In contrast, the milder remodeling effect of MLCT-C further corroborates that structured lipids, while providing anti-obesity benefits, can better maintain the relative stability of the core microecosystem [62].

#### 3.11.3. Functional Annotation of the Gut Microbiota in Mice

**Figure 13:**
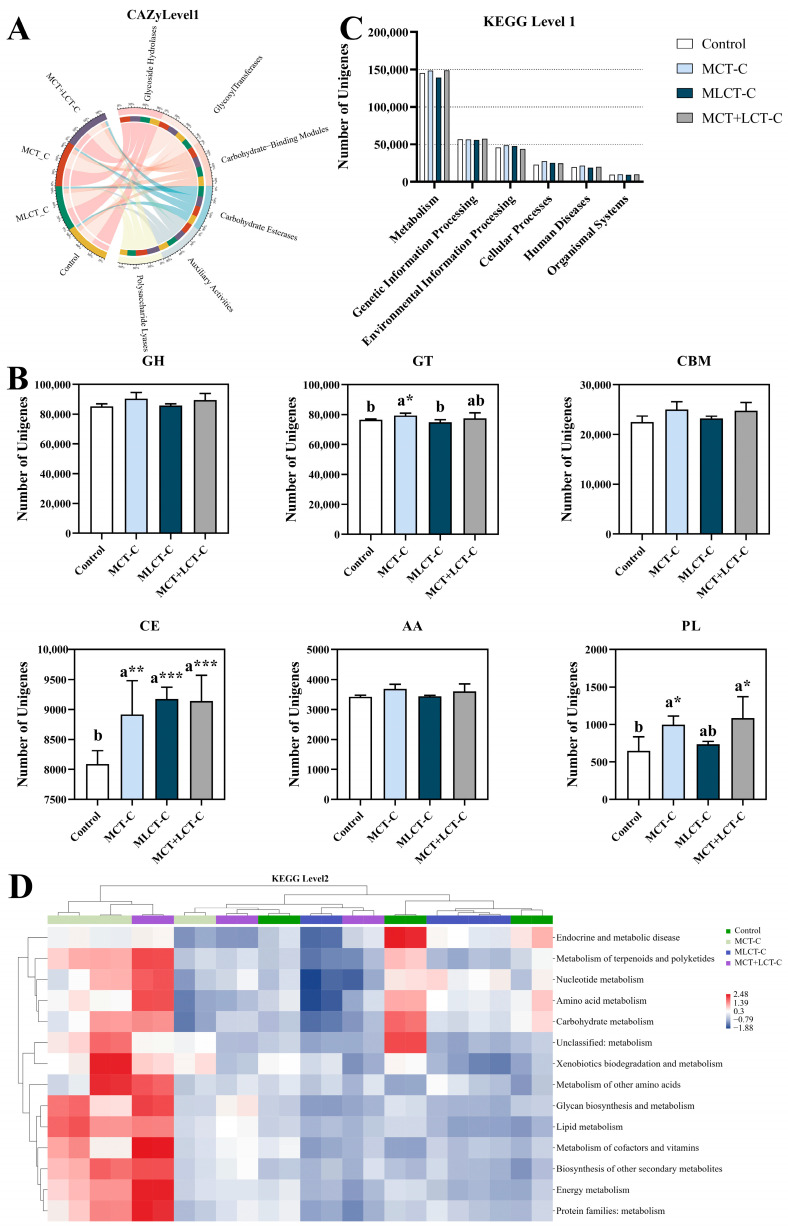
Functional annotation of the mouse gut microbiota. (**A**) CAZy Level 1 classification; (**B**) CAZy Level 2 classification; (**C**) KEGG functional enrichment at Level 1; (**D**) KEGG functional enrichment at Level 2. Lowercase letters (a and b) in (**B**) indicate significant intergroup differences (*p* < 0.05). Asterisks in (**B**) denote statistical significance relative to the control group (* *p* < 0.05, ** *p* < 0.01, *** *p* < 0.001).

Based on Unigene-derived protein sequences, functional annotation was performed using the DIAMOND algorithm against the CAZy and KEGG databases. Annotation information for each Unigene was obtained from both databases. Subsequently, by integrating Unigene abundance profiles, the functional composition at different hierarchical levels was quantified for each sample. Intergroup differential analysis was then conducted, and the results are presented in Figure 13.

Statistical analysis of CAZy profiles across the four groups revealed significant differences at the levels of Glycosyl Transferases (GT), Polysaccharide Lyases (PL), Carbohydrate Esterases (CE), Auxiliary Activities (AA), and Carbohydrate-Binding Modules (CBM) (*p* < 0.05). Among these, GT, CE, and PL exhibited highly significant differences (*p* < 0.01), whereas no significant difference was observed at the level of Glycoside Hydrolases (GH) (*p* > 0.05).

Multiple comparison analysis demonstrated that, at the GT level, both the MLCT-C group and the control group were significantly lower than the MCT-C group, while no significant differences were observed among these groups and the MCT+LCT-C group. Although significant overall differences were detected at the CBM and AA levels, pairwise comparisons did not reveal clear intergroup differences, suggesting that the observed variation was primarily driven by cumulative effects across multiple groups rather than distinct pairwise contrasts. CE level exhibited the most pronounced differences, with the control group showing markedly lower levels than the MCT-C group and substantially lower levels than both the MLCT-C and MCT+LCT-C groups. At the PL level, the control group was significantly lower than the MCT-C and MCT+LCT-C groups, whereas no significant differences were observed between the MLCT-C group and the other groups.

Except for CE, regardless of statistical significance, the CAZy functional profiles of the MLCT-C group consistently resembled those of the control group most closely, whereas the MCT-C and MCT+LCT-C groups generally exhibited elevated levels. This pattern was particularly evident at the GT and PL levels. These findings indicate that the functional structure of the gut microbiota in MLCT-C-treated mice is more similar to that of the control group, suggesting that the combined MLCT-C intervention may mitigate the adverse effects induced by a high-fat diet.

In addition, the dispersion of functional profiles was lower in the control and MLCT-C groups, whereas greater variability was observed in the MCT-C and MCT+LCT-C groups. This may indicate that the gut microbiota functional composition in the MLCT-C group is more stable, with individual samples exhibiting more consistent responses to the intervention.

Currently, studies focusing on MLCTs rarely address CAZy level functional alterations in the gut microbiota, so the underlying molecular mechanisms remain to be elucidated.

At the KEGG Level 1 annotation, metabolic pathways were the predominant functional category across all groups, which is consistent with previous findings reported by Yuan [62]. Further analysis at KEGG Level 2 revealed that samples within the MLCT-C group clustered tightly and were frequently grouped within the same branch as the control group, indicating similar overall functional profiles and relatively lower expression of associated functional genes. In contrast, the MCT-C group tended to form distinct clusters, clearly separated from other groups, suggesting substantial functional divergence. Meanwhile, the MCT+LCT-C group exhibited an interspersed distribution with other groups, without clear separation, indicating an unstable functional profile. These observations further support that MLCT-C intervention may stabilize the functional structure of the gut microbiota and attenuate the perturbations induced by a high-fat diet.

**Figure 14:**
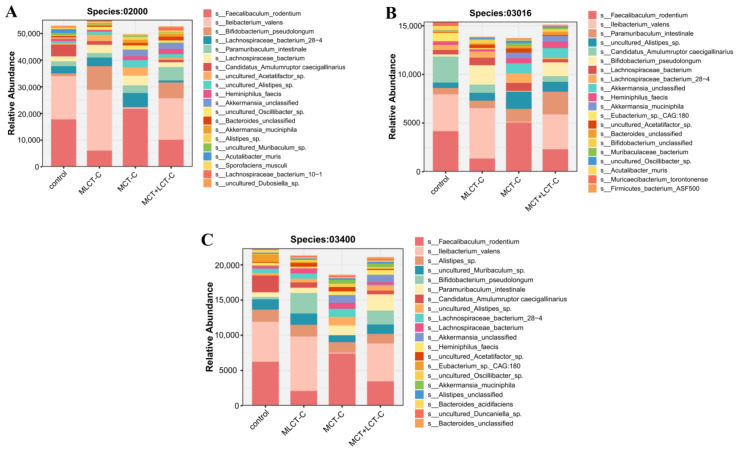
Species and functional contribution analysis of the gut microbiota: (**A**) Species and functional contribution analysis of KEGG 02000; (**B**) Species and functional contribution analysis of KEGG 03016; (**C**) Species and functional contribution analysis of KEGG 03400.

Comprehensive analysis of KEGG functional pathways in the gut microbiota showed that the three most abundant pathways were 02000 (Transporters), 03016 (RNA Transport), and 03400 (DNA Repair and Recombination Proteins). Combined with gut microbiota species abundance analysis, the three treatment groups exhibited a consistent trend of pathway activation in transmembrane transport of substances, modulation of gene expression and preservation of genetic stability compared with the control group. Figure 14 presents the top 20 representative species contributing most significantly to these three major pathways. Notably, the observed alterations were predominantly driven by the primary functional contributors, *Faecalibaculum rodentium* and *Bifidobacterium pseudolongum*.

*F. rodentium* was the main functionally contributing bacterium across the three pathways (02000, 03016, 03400). The MCT-C group exhibited significantly higher *F. rodentium* abundance than the control group, while the MLCT-C and MCT+LCT-C groups showed significantly lower abundance. This bacterium is rich in ABC and MFS-type transport systems, RNA-binding proteins, and DNA repair enzyme genes such as *RecA* and *MutS/L* [76,77,78]. The increase in the MCT-C group suggests that this treatment promoted its functions in carbohydrate transport, protein translation, and maintenance of genetic stability, reflecting a stress adaptation characteristic of high metabolism and high repair. In contrast, its decrease in the MLCT-C and MCT+LCT-C groups may be related to ecological competition induced by high-energy substrates or differences in substrate preference—specifically, the metabolic activity of *F. rodentium* was inhibited under high-fat conditions.

In contrast, *B. pseudolongum* exhibited an opposite trend in the three pathways compared with *F. rodentium*. This bacterium showed a high functional contribution in the control group, while its abundance was significantly decreased in the MCT-C group, and it ceased to function as the primary contributing genus *B. pseudolongum* was shown to be rich in genes related to substrate transport and carbohydrate metabolism for the utilization of host-fermentable carbon sources (such as oligosaccharides and host glycans) in its genome. It also possessed genetic characteristics targeting exogenous DNA invasion and repair mechanisms, such as restriction/modification systems and DNA recombination repair pathways [79,80]. The decrease in its abundance may indicate that MCT-C treatment inhibited its metabolic activity, or its niche was partially replaced by other microbiota due to the conversion of energy substrates.

Although the overall trends of the three pathways were consistent, the two core bacteria exhibited opposite response directions, reflecting the metabolic structure reorganization of the microbiota under the stimulation of exogenous energy substrates. *F. rodentium* showed functional activation in the MCT-C group, acting as a rapid-response bacterium in the high-energy metabolic environment. In contrast, *B. pseudolongum* decreased under the same conditions, which may reflect its sensitivity to changes in substrate structure or metabolic flux. This differential response suggests that the three interventions altered the functional division of labor of the microbiota in terms of substance transport, RNA metabolism, and DNA repair by regulating intestinal carbon sources and energy flux, thereby forming a new metabolic pattern at the macro level.

## 4. Conclusions

This study investigated the combined effects of different lipid formulations (MCTs, MLCTs, and MCT+LCT mixtures) with natural carotenoids (β-carotene and α-carotene) on HFD-induced obese mice, revealing their differential impacts on metabolic outcomes. The results indicated that the MCT-C diet demonstrated the most significant effects on body weight control and reduction in fat deposition, possibly through reduced appetite, altered energy metabolism, and shifts in gut microbiota composition. These advantages, however, could come with a degree of hepatic strain. In contrast, the MLCT-C and MCT+LCT-C diets showed similar influences on macro-metabolic phenotypes. Nevertheless, MLCT-C exhibited greater advantages in maintaining gut microbiota homeostasis and more effectively modulating microbial composition, while MCT+LCT-C led to more pronounced adipocyte hypertrophy.

Different lipid types distinctively shape the gut microbiota, which in turn modulates host metabolism. The weight-reducing effect of MCT-C correlated with increased *Akkermansia* abundance and activation of specific metabolic pathways. In contrast, MLCT-C showed unique benefits in maintaining overall microbial balance. These findings suggest that the structure of dietary lipids governs metabolic outcomes not only directly but also by channeling their effects through the gut microbiota. This provides a new direction for future research on the causal mechanisms linking gut microbiota and metabolic phenotypes.

In summary, MLCT-C provides metabolic benefits comparable to those of MCT+LCT-C while demonstrating superior gut microbiota modulation capability. Although MCT-C significantly promotes weight loss, its potential burden on the liver warrants attention. The intervention strategy of combining structured lipids with natural bioactive compounds in this study provides an important foundation for subsequent clinical research. To further optimize this approach for precision nutrition, comprehensively characterizing the dynamic in vivo absorption kinetics and tissue deposition of the co-delivered carotenoids represents an essential next step.

## Figures and Tables

**Table 1:** Macronutrient composition of the experimental diets.

Macronutrient Composition	Low-Fat Diet Group	MCT-C Group	MLCT-C Group	MCT+LCT-C Group
Protein (%)	19.24	23.65	23.65	23.65
Carbohydrate (%)	66.82	40.85	40.85	40.85
Fat (%)	4.27	23.61	23.61	23.61

**Table 2:** Ingredients of the experimental diets.

Ingredients	Low-Fat Diet Group	MCT-C Group	MLCT-C Group	MCT+LCT-C Group
Casein (g)	233.05	233.06	233.06	233.06
L-Cystine (g)	3.50	3.50	3.50	3.50
Corn Starch (g)	526.93	84.83	84.83	84.83
Maltodextrin (g)	87.39	116.53	116.53	116.53
Sucrose (g)	206.02	206.02	206.02	206.02
Cellulose (g)	58.26	58.26	58.26	58.26
Carotenoids (mg)	0.00	150.00	150.00	150.00
Soybean Oil (g)	29.13	0.00	0.00	0.00
Lard (g)	23.31	0.00	0.00	0.00
MLCTs (g)	0.00	0.00	235.97	0.00
MCTs (g)	0.00	235.97	0.00	0.00
MCTs + LCTs (g)	0.00	0.00	0.00	235.97
Mineral Mix S10026B (g)	58.26	58.26	58.26	58.26
Vitamin Mix V10001C (g)	1.17	1.17	1.17	1.17
Choline Bitartrate (g)	2.33	2.33	2.33	2.33
Total (g)	1229.35	1000.15	1000.15	1000.15

**Table 3:** Fatty acid composition of the experimental oils.

	Sample		
Fatty Acid	MCTs (%)	MLCTs (%)	MCTs + LCTs (%)
C8:0	58.03 ± 0.09	9.69 ± 0.07	9.52 ± 0.07
C10:0	40.45 ± 0.05	6.47 ± 0.02	6.67 ± 0.02
C11:0	0.06 ± 0.02	0.00 ± 0.00	0.01 ± 0.00
C12:0	0.53 ± 0.03	0.03 ± 0.00	0.09 ± 0.00
C14:0	0.01 ± 0.02	0.06 ± 0.00	0.06 ± 0.00
C16:0	0.12 ± 0.02	8.50 ± 0.00	8.00 ± 0.00
C16:1C	0.00 ± 0.00	0.07 ± 0.01	0.07 ± 0.00
C17:0	0.00 ± 0.00	0.07 ± 0.00	0.07 ± 0.00
C17:1	0.00 ± 0.00	0.02 ± 0.03	0.04 ± 0.00
C18:0	0.02 ± 0.00	3.71 ± 0.01	3.24 ± 0.01
C18:1C	0.23 ± 0.01	20.82 ± 0.01	21.36 ± 0.01
C18:2T	0.00 ± 0.00	0.22 ± 0.00	0.14 ± 0.00
C18:2C	0.50 ± 0.01	43.18 ± 0.05	43.54 ± 0.05
C20:0	0.00 ± 0.00	0.35 ± 0.01	0.27 ± 0.00
C18:3-9C,12T,15T + C18:3-9C,12C,15T hr *	0.00 ± 0.00	0.21 ± 0.00	0.08 ± 0.00
C18:3-9T,12C,15C hr	0.00 ± 0.00	0.09 ± 0.13	0.07 ± 0.00
C20:1C hr	0.00 ± 0.00	0.08 ± 0.11	0.14 ± 0.00
C18:3-9C,12C,15C hr	0.01 ± 0.00	5.73 ± 0.29	6.19 ± 0.31
C22:0 hr	0.02 ± 0.00	0.38 ± 0.01	0.41 ± 0.00
C23:0 hr	0.00 ± 0.00	0.03 ± 0.01	0.04 ± 0.00
C24:0 hr	0.01 ± 0.01	0.12 ± 0.00	0.14 ± 0.00
C16:1	0.00 ± 0.00	0.08 ± 0.01	0.09 ± 0.00
C18:1	0.23 ± 0.01	20.85 ± 0.01	21.39 ± 0.01
C18:2	0.50 ± 0.01	43.4 ± 0.04	43.68 ± 0.05
C18:3	0.01 ± 0.00	6.03 ± 0.15	6.33 ± 0.31
SAFA	99.26 ± 0.02	29.49 ± 0.06	28.57 ± 0.10
MUFA	0.23 ± 0.01	21.00 ± 0.13	21.60 ± 0.01
PUFA	0.51 ± 0.01	49.47 ± 0.18	50.04 ± 0.27
TRANS	0.00 ± 0.00	0.57 ± 0.13	0.33 ± 0.00

Note: Data are expressed as mean ± SD (*n* = 2). * Co-eluted with minor component(s) (annotated as “hr” in the chromatogram). Abbreviations: SAFA, saturated fatty acids; MUFA, monounsaturated fatty acids; PUFA, polyunsaturated fatty acids; TRANS, trans fatty acids.

**Table 4:** The physicochemical characteristics of oils used in this experiment.

Parameters	MCTs	MLCTs	MCTs + LCTs
Acid value (mg KOH/g)	0.025 ± 0.007	0.265 ± 0.020	0.074 ± 0.006
Peroxide value (mmol/kg)	ND	0.510 ± 0.010	0.657 ± 0.018
Moisture content (%)	0.028 ± 0.020	0.015 ± 0.000	0.023 ± 0.005

Note: Data are expressed as mean ± SD (*n* = 2). “ND” indicates not detected (below the detection limit of the method).

**Table 5:** Adonis analysis of beta diversity in the mouse gut microbiota.

Comparison	R^2^	F-Value	*p*-Value
MLCT-C vs. MCT-C	0.598	14.91	0.005
MCT-C vs. control	0.555	12.47	0.001
MCT+LCT-C vs. MCT-C	0.528	11.19	0.001
MCT+LCT-C vs. control	0.435	7.69	0.003
MLCT-C vs. control	0.434	7.66	0.002
MLCT-C vs. MCT+LCT-C	0.307	4.43	0.012

## Data Availability

The original contributions presented in the study are included in the article/Supplementary Materials; further inquiries can be directed to the corresponding authors.

## Supplementary Materials

The following supporting information can be downloaded at: https://www.mdpi.com/article/10.3390/foods15132285/s1, Figure S1: Gas chromatogram of MLCTs; Figure S2: Gas chromatogram of MCTs + LCTs.

## Abbreviations

The following abbreviations are used in this manuscript:

AA	Auxiliary activities
ABC	ATP-binding cassette
acetyl-CoA	Acetyl coenzyme A
ALT	Alanine aminotransferase
ANOVA	Analysis of variance
ANOSIM	Analysis of similarities
AST	Aspartate aminotransferase
ATGL	Adipose triglyceride lipase
BAT	Brown adipose tissue
BCO1	Beta-carotene oxygenase 1
BMI	Body mass index
bp	Base pairs
CAZy	Carbohydrate-active enzymes database
CBM	Carbohydrate-binding modules
CDSs	Coding sequences
CE	Carbohydrate esterases
DIO	Diet-induced obesity
DNA	Deoxyribonucleic acid
dsDNA	Double-stranded DNA
FAC	Fatty acid composition
FAMEs	Fatty acid methyl esters
F/B ratio	*Bacillota*/*Bacteroidota ratio*
FID	Flame ionization detector
FMT	Fecal microbiota transplantation
GB	Guobiao/National Standard of China
GH	Glycoside hydrolases
GT	Glycosyl transferases
H&E	Hematoxylin and eosin
HDL-C	High-density lipoprotein cholesterol
HFD	High-fat diet
HPLC	High-performance liquid chromatography
HSL	Hormone-sensitive lipase
IQR	Interquartile range
KEGG	Kyoto Encyclopedia of Genes and Genomes
LCFAs	Long-chain fatty acids
LCTs	Long-chain triglycerides
LDL-C	Low-density lipoprotein cholesterol
LEfSe	Linear discriminant analysis Effect Size
LFD	Low-fat diet
MAG	Monoacylglycerols
MCFAs	Medium-chain fatty acids
MCTs	Medium-chain triglycerides
MFS	Major facilitator superfamily
MLCTs	Medium- and long-chain triglycerides
MPOB	Malaysian Palm Oil Board
MRI	Magnetic resonance imaging
MUFA	Monounsaturated fatty acids
NCBI	National Center for Biotechnology Information
ND	Not detected
NEFA	Non-esterified fatty acids
NMDS	Non-metric multidimensional scaling
NR	Non-Redundant
ORFs	Open reading frames
OTU	Operational Taxonomic Unit
PCA	Principal component analysis
PCoA	Principal coordinate analysis
PCR	Polymerase chain reaction
PKA	cAMP-dependent protein kinase A
PL	Polysaccharide lyases
PNS	*Panax notoginseng saponins*
PPAR-α	Peroxisome proliferator-activated receptor alpha
PPAR-γ	Peroxisome proliferator-activated receptor gamma
PUFA	Polyunsaturated fatty acids
RAR/RXR	Retinoic acid receptor/Retinoid X receptor
RO	Rice oil
SAFA	Saturated fatty acids
SAT	Subcutaneous adipose tissue
SCFAs	Short-chain fatty acids
SD	Sprague Dawley; standard deviation
SEM	Standard error of the mean
SPF	Specific pathogen-free
SPSS	Statistical Product and Service Solutions
TC	Total cholesterol
TG	Triglycerides
TRANS	Trans fatty acids
VAT	Visceral adipose tissue
WAT	White adipose tissue
WHO	World Health Organization
