# Supplementary material for: Effects of Medium-Chain Versus Medium- and Long-Chain Triglycerides, Combined with Carotenoids, in a High-Fat Diet on Obese Mice
Source: Foods. 2026 Jun 25;15(13):2285. doi: 10.3390/foods15132285 (PMC13361576; doi:10.3390/foods15132285)
Supplement: Supplementary file 1 [file foods-15-02285-s001.zip › Figures S1 and S2.pdf]

Figures S1 and S2

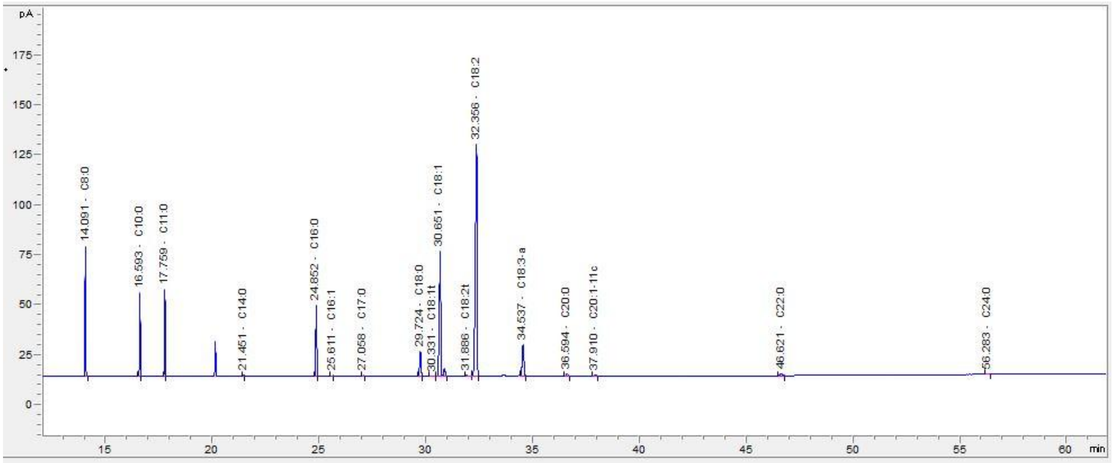

Figure S1. Gas chromatogram of MLCTs

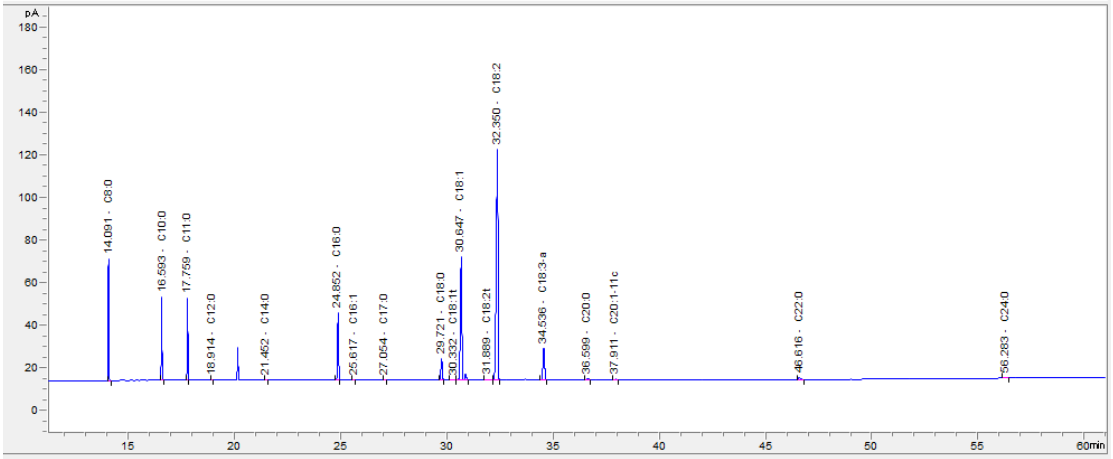

Figure S2. Gas chromatogram of MCTs+LCTs

Disclaimer/Publisher's Note: The statements, opinions and data contained in all publications are solely those of the individual author(s) and contributor(s) and not of MDPI and/or the editor(s). MDPI and/or the editor(s) disclaim responsibility for any injury to people or property resulting from any ideas, methods, instructions or products referred to in the content.
